# Multicomponent versus domino reactions: One-pot free-radical synthesis of β-amino-ethers and β-amino-alcohols

**DOI:** 10.3762/bjoc.11.10

**Published:** 2015-01-15

**Authors:** Bianca Rossi, Nadia Pastori, Simona Prosperini, Carlo Punta

**Affiliations:** 1 Department of Chemistry, Materials, and Chemical Engineering "G. Natta" - Politecnico di Milano, Via Leonardo da Vinci 32, I-20131 Milano, Italy; Fax (+39)0223993180

**Keywords:** aminoalcohols, free-radical addition, imine, multicomponent reaction, titanium salts

## Abstract

Following an optimized multicomponent procedure, an aryl amine, a ketone, and a cyclic ether or an alcohol molecule are assembled in a one-pot synthesis by nucleophilic radical addition of ketyl radicals to ketimines generated in situ. The reaction occurs under mild conditions by mediation of the TiCl_4_/Zn/*t*-BuOOH system, leading to the formation of quaternary β-amino-ethers and -alcohols. The new reaction conditions guarantee good selectivity by preventing the formation of secondary products. The secondary products are possibly derived from a competitive domino reaction, which involves further oxidation of the ketyl radicals.

## Introduction

Multicomponent reactions (MCRs) represent a green approach towards the synthesis of polyfunctionalized molecules by promoting multiple bond-forming mechanisms in a one-pot synthesis [1–7]. Likewise, the development of radical-mediated, C–C bond-forming transformations has also attracted increasing interest. These protocols usually afford good selectivity, are highly compatible with common functional groups, and often require mild operative conditions [8–12].

During the course of our ongoing investigation of the role of titanium salts in mediating selective radical transformations [13–14], we have developed new, simple protocols for the synthesis of complex organic compounds through nucleophilic radical addition to imines generated in situ [15]. Several recent contributions to the field clearly illustrate how the free-radical approach can be considered a valuable alternative to classical ionic protocols [16–23].

In this context, we succeeded in combining the advantages of the radical synthetic route with the high added value of a multicomponent approach. In fact, our procedure requires neither the preformation of imines nor anhydrous media, due to the coordinating effect of titanium salts, which promote the one-pot synthesis of amino derivatives according to a classical MCR.

**Scheme 1:**
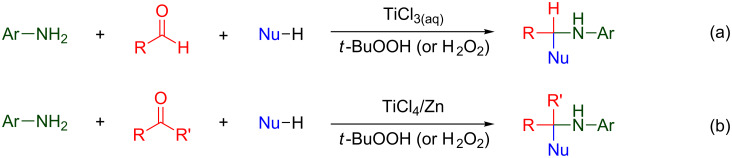
Nucleophilic radical addition to imines mediated by titanium salts.

The basic approach consists of the simple mixing of an aniline, an aldehyde or ketone, and a suitable source of nucleophilic radicals (Nu–H = formamide [24–25], methanol [26–27], ethers [28]), in the presence of titanium chlorides, and slowly dropping a solution of hydroperoxide (H_2_O_2_ or *tert*-butyl hydroperoxide (*t*-BuOOH)) into the reaction medium (Scheme 1).

Aqueous TiCl_3_ proved particularly effective for promoting the formation and functionalization of aldimines (Scheme 1, entry a), but was necessarily replaced with the redox TiCl_4_ (CH_2_Cl_2_)/Zn system when operating in the presence of ketones (Scheme 1, entry b), as the corresponding ketimines were more sensitive to the aqueous medium [25,27].

When we first tried to extend this procedure to the addition of alcohols other than methanol, a side reaction competed with the multicomponent mechanism, that is, the oxidation of the ketyl radical derived from the alcohol to the corresponding aldehyde. This transformation significantly affected the selectivity in the desired products.

**Scheme 2:**
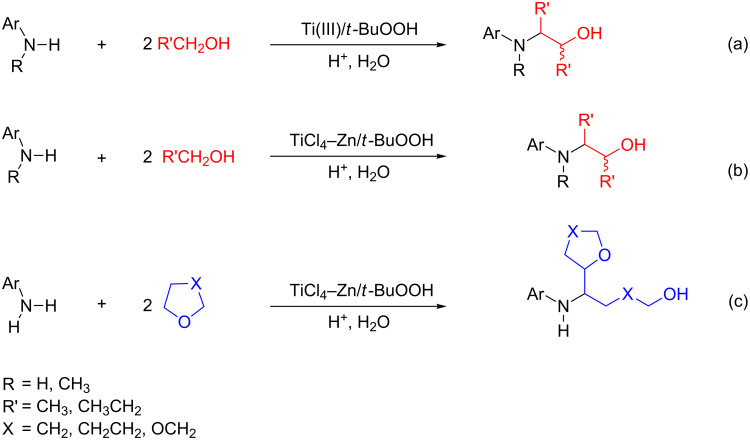
Radical domino approach to the synthesis of β-aminoacohols triggered by titanium salts.

Taking advantage of this secondary process, we performed a domino reaction, where the alcohol served as the source of both the nucleophilic radical and the aldehyde necessary for the in situ formation of the aldimine (Scheme 2, entry a) [29]. Surprisingly, the same reaction was even more efficient when performed in the presence of the Ti(IV)/Zn/*t*-BuOOH system (Scheme 2, entry b). Under the latter conditions, the domino approach was also successfully extended to cyclic ethers, promoting the consecutive electrophilic amination to imines through a ring opening of the ether (Scheme 2, entry c) [30].

Even though it is interesting by itself, this domino reaction limits the general approach towards the synthesis of a wider range of β-amino-alcohols and ethers. The importance of these derivatives is well-documented. They are the key components of several synthetic and natural molecules, exhibiting signiﬁcant biological and chemical properties [31–35]. For this reason, efforts are continuously being devoted to the development of new, efficient and simple synthetic procedures [36–43]. Herein we report the results of our in depth investigation to allow for the one-pot multicomponent addition of alcohols and ethers to ketimines generated in situ, by inhibition of the domino reaction.

## Results and Discussion

The optimization of the multicomponent approach was conducted by the one-pot assembly of *p*-toluidine, tetrahydrofuran (THF) and acetone in the presence of the TiCl_4_/Zn system as a model reaction. The reaction occurred when adding a solution of *t-*BuOOH in THF dropwise into the reaction mixture. The different process conditions, resulting in the synthesis of **1a**, are reported in Table 1.

**Table 1:** Optimization of reaction conditions: multicomponent versus domino reaction.

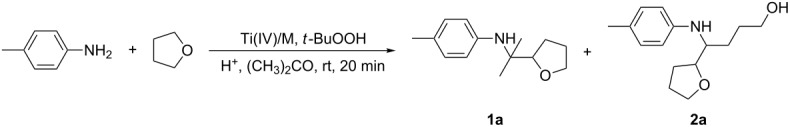						

Entry	TiCl_4_–M	THF (mL)	Acetone (mL)	CH_2_Cl_2_ (mL)	**1** (Yield, %^a^)	**2** (Yield, %^a^)

1	M = Zn	10	–	–	–	84^b^
2	M = Zn	10	0.37 (5 mmol)	–	66	11
3	M = Zn	–^c^	10	–	63	–
4	M = Zn	–^c^	1	5	70	–
**5**	**M = Zn**	**–^c^**	**0.37 (5 mmol)**	**5**	**77**	–
6	M = Mn	–^c^	0.37 (5 mmol)	5	45	–
7	M = Fe	–^c^	0.37 (5 mmol)	5	–	–
8	–	–^c^	0.37 (5 mmol)	5	–	–
9	M = Zn^d^	–^c^	0.37 (5 mmol)	5	–	–

^a^Yield determined by ^1^H NMR spectroscopy with acetophenone added as an internal standard to the crude reaction mixture after work-up. ^b^Data taken from [30]. ^c^THF was added dropwise diluted in a 80 wt % solution of *t*-BuOOH. ^d^In the absence of TiCl_4_.

**Scheme 3:**
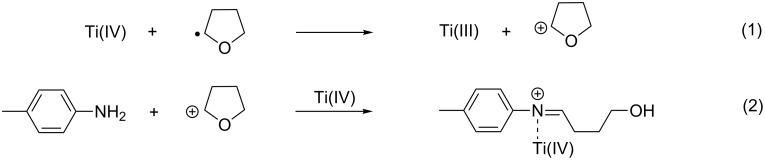
Competitive imine formation from THF.

As previously described, the use of THF as solvent in the absence of acetone led to the selective formation of the domino product, **2a**, in a high yield (Table 1, entry 1). While the simple addition of acetone to the reaction mixture promoted the production of the desired **1a**, the selectivity of the process was poor due to the concomitant formation of **2a** (Table 1, entry 2). We ascribed this result to the high volume of THF, which competes with the ketone in the formation of imines (Scheme 3).

Indeed, by simply using acetone as a solvent, and adding the minimum quantity of THF with *t-*BuOOH necessary, we succeeded in suppressing the formation of **2a**, achieving an analogous yield of **1a**, but with complete selectivity (Table 1, entry 3). Nevertheless, this solution hindered the ultimate goal of developing a general procedure for the addition of ketyl radicals to a wide range of ketimines. Other ketones besides acetone have limited applications as solvents, both due to economic issues and with regards to their limited solubilization properties. For this reason, we verified the possibility of using dichloromethane as a solvent by decreasing the amount of acetone down to 1 mL (Table 1, entry 4), and to 5 mmol (Table 1, entry 5), achieving even higher yields (up to 77%).

**Scheme 4:**
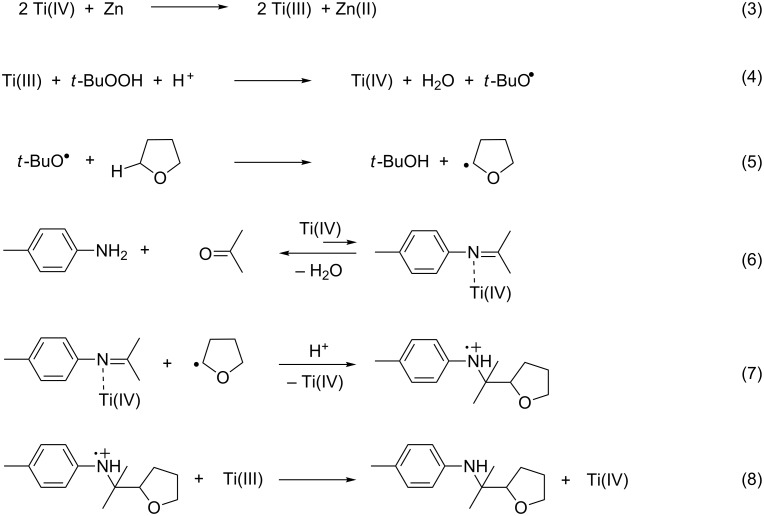
Reaction mechanism.

The possible reaction mechanism, analogous to that described in previous works, is reported in Scheme 4. Ti(III), generated in situ from the redox process between Ti(IV) and Zn (Scheme 4, reaction 3), reacts in turn with *t-*BuOOH, leading to the corresponding alkoxy radical (Scheme 4, reaction 4). The latter undergoes hydrogen abstraction from the C–H bond of THF in the α-position of the oxygen (Scheme 4, reaction 5). This promotes the formation of a carbon-centered nucleophilic radical, which in turn quickly adds to the activated carbon of the imine generated in situ. In this context, Ti(IV) not only initiates the redox process but, being a Lewis acid, also coordinates the nitrogen atom, promoting the formation of the imine under non-anhydrous conditions (Scheme 4, reaction 6) and rendering the carbon atom in the imine moiety more electrophilic. This strong polar effect is the real driving force of the overall process, making the imine more reactive towards nucleophilic addition by the ketyl radicals (Scheme 4, reaction 7), and consequently shifting the equilibrium of reaction 6 towards the right. The catalytic cycle is concluded with the final reduction of the iminium radical intermediate by Ti(III) (Scheme 4, reaction 8).

As expected, in the absence of TiCl_4_ (Table 1, entry 8) or Zn (Table 1, entry 9), no conversion was observed. Moreover, we also replaced Zn with other metals, namely Mn (Table 1, entry 6) and Fe (Table 1, entry 7), with the aim of further improving the efficiency of the process, however, both cases resulted in the lowest conversion observed. With the optimized conditions, we proceeded exploring the scope of the reaction and the results are reported in Table 2.

**Table 2:** Aminoalkylation of ethers and alcohols.

Entry	Aromatic amine	Ketone	Ether/alcohol	Product	Yield, %^a^ (yield, %)^b^

1	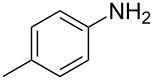	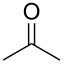	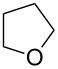	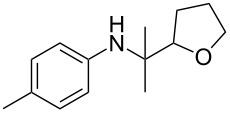 **1a**	77 (63)
2	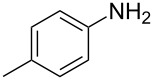	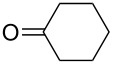	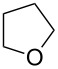	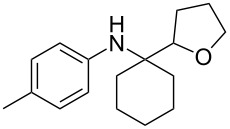 **1b**	75 (64)
3	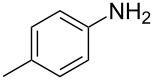	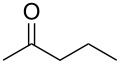	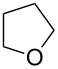	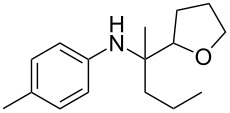 **1c**	64 (58)
4	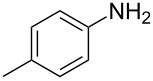	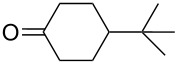	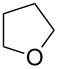	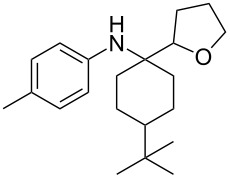 **1d**	71 (49)
5	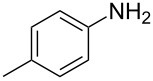	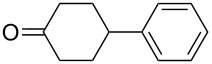	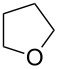	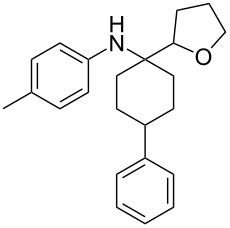 **1e**	60 (40)
6	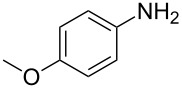	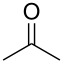	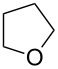	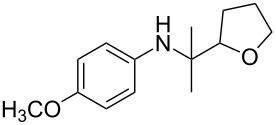 **1f**	60 (57)
7	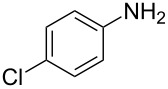	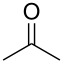	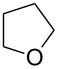	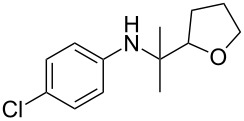 **1g**	75 (69)
8	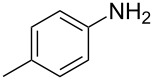	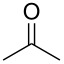	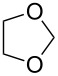	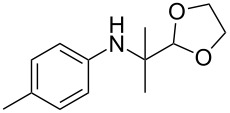 **1h**	26 (18)
9	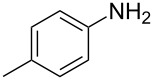	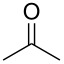	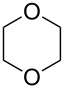	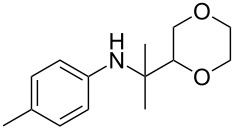 **1i**	39 (30)
10	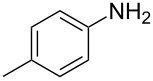	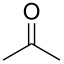	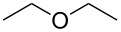	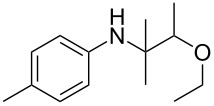 **1j**	32 (25)
11	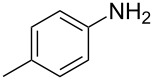	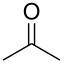	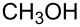	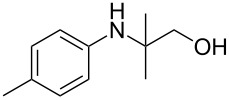 **1k**	75 (68)^c^
12	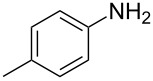	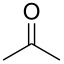	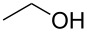	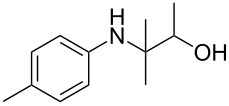 **1l**	70 (64)^d^
13	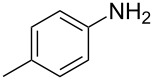	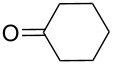	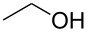	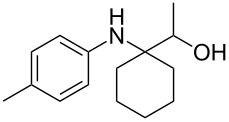 **1m**	81 (75)^d^
14	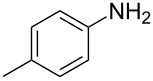	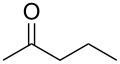	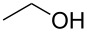	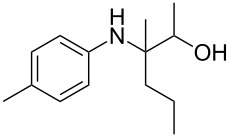 **1n**	50 (41)^e^
15	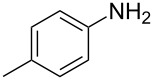	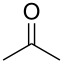	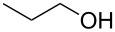	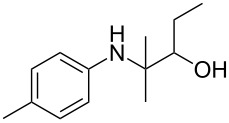 **1°**	86 (61)
16	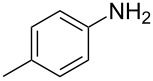	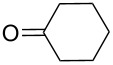	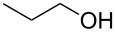	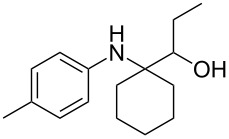 **1p**	81 (60)

^a^Yield determined by ^1^H NMR spectroscopy with acetophenone added as an internal standard to the crude reaction mixture after work-up. ^b^Yield of isolated products based on the starting amines. ^c^Data taken from [27]. ^d^Alcohol was used as solvent. ^e^9% of domino reaction product **2b** was observed.

The addition of THF to ketimines generated in situ afforded the desired products **1a–1g** in good yields, both starting from different acyclic and cyclic ketones (Table 2, entries 1–5) and from different amines (Table 2, entry 6 and entry 7). As already observed in our previous reports, secondary anilines were not converted under our operative conditions, likely due to an enhanced steric hindrance in the formed ketimine. The same reaction occurred by replacing THF with 1,3-dioxolane, 1,4-dioxane, and diethyl ether, affording the corresponding products **1h**, **1i**, and **1j**, respectively, with complete selectivity but lower yields.

**Scheme 5:**
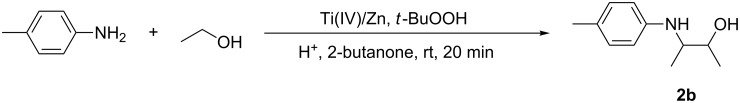
Domino reaction in the presence of ethanol.

Finally, for the first time, the aminoalkylation was also extended to alcohols other than methanol (Table 2, entry 11), namely ethanol and propanol, achieving high yields and high selectivity in all cases while successfully inhibiting the domino side-reaction (Table 2, entries 12–16). Although ethanol could be used as a solvent without substitution with CH_2_Cl_2_ in the reaction medium, when operating in the presence of propanol, it was necessary to adopt the same optimized conditions of THF. Only when reacting *p*-toluidine with 2-butanone in ethanol, a 9% of domino byproduct **2b** (Scheme 5) was observed.

## Conclusion

We have reported optimized conditions for the selective free radical nucleophilic addition of ethers and alcohols to ketimines generated in situ under non-anhydrous conditions. The protocol consists of a three-component, one-pot reaction involving an aromatic amine, a ketone, and the source of the ketyl radical. The new conditions prevent the undesired domino side-reaction from occurring, whereby the ketyl radical undergoes fast oxidation and generates new carbonyl derivatives, which in turn competes with ketones in the formation of imines.

Titanium salts play a key role, both favoring the formation of ketimines and promoting their reactivity towards the nucleophilic addition. The general applicability of the reaction has been demonstrated by reacting different aromatic amines, ketones, ethers and alcohols. This approach provides a new protocol for the simple and convenient synthesis of a wide range of amino derivatives, under mild conditions without requiring isolation and purification of intermediates, according to a classical multicomponent reaction.

## Experimental

All materials were purchased from commercial suppliers and used without further purification. All reactions were performed at room temperature (20 °C) under nitrogen atmosphere. The following solutions were used: 1 M solution of TiCl_4_ in CH_2_Cl_2_ and 80 wt % solution of *t*-BuOOH. NMR spectra were recorded at 400 MHz for ^1^H and 100 MHz for ^13^C. ESIMS was performed with an Esquire 3000 plus ion-trap mass spectrometer equipped with an ESI source. Tandem mass spectra were obtained by CID with helium collision gas after isolation of the precursor ion. Mass spectra were additionally measured with a GC–MS instrument, using a gas chromatograph equipped with an SBP-1 fused silica column (30 m × 0.2 mm i.d., 0.2 μm film thickness) and helium as carrier gas. The IR spectra were obtained by a Varian 640 high-performance FTIR spectrometer. The melting points were measured on a Büchi 535 apparatus. Flash column chromatography was performed by using 40–63 μm silica gel packing; the eluent was chosen in order to move the desired components to *R*_f_ 0.35 on analytical TLC.

### General procedure for β-radical aminoalkylation of ethers

In a manner similar to the procedures of [25] and [27], a homogeneous solution of CH_2_Cl_2_ (5 mL), containing an aryl amine (2 mmol), a cyclic or acyclic ketone (5 mmol), and TiCl_4_ (2.5 mL of 1 M solution in CH_2_Cl_2_, 2.5 mmol), was stirred at room temperature under N_2_ atmosphere. After approximately 30 min, Zn powder (300 mg, ca. 5 mmol) was suspended in the reaction medium, and an aqueous 80 wt % solution of *t*-BuOOH (10 mmol, ca. 1.2 mL), diluted in the ether of choice (9 mL), was added dropwise. The reaction has the appearance of a titration experiment and proceeds with periodic changes of color from orange to violet. *t*-BuOOH was added until a pale orange color was barely maintained. At this point, a second portion of TiCl_4_ (2.5 mmol) and of Zn powder (300 mg, ca. 5 mmol) were added and the dropwise addition of *t*-BuOOH was continued until the solution reached a stable orange color. The co-solvent was removed under vacuum and the reaction was quenched with H_2_O (5 mL). A 30% aqueous NH_3_ solution was added until a basic pH was reached (a white precipitate due to Ti(IV) hydroxide was observed). The resulting aqueous solution was extracted with EtOAc (3 × 50 mL), and the combined organic layers were dried over Na_2_SO_4_ and concentrated in vacuum. Purification of the crude residue by flash chromatography afforded aminoalkylated ethers as the last eluting products. Yields of isolated products are based on the starting amine.

### General procedure for β-radical aminoalkylation of alcohols

A homogeneous solution of ethanol (5 mL, for products **1l**, **1m**, and **1n**) or CH_2_Cl_2_ (5 mL, for products **1o** and **1p**), containing an arylamine (2 mmol), a cyclic or acyclic ketone (5 mmol), and TiCl_4_ (2.5 mL of 1 M solution in CH_2_Cl_2_, 2.5 mmol), was stirred at rt under N_2_ atmosphere. After approximately 30 min, Zn powder (300 mg, ca. 5 mmol) was suspended in the reaction medium, and an aqueous 80 wt % solution of *t*-BuOOH (10 mmol, ca. 1.2 mL), diluted in the alcohol of choice (9 mL), was added dropwise. The reaction procedure and work-up are analogous to that of the β-radical aminoalkylation of ethers.

## Supporting Information

File 1 Analytical data and ^1^H and ^13^C NMR spectra of compounds **1a–p**.
