# Supplementary material for: Multicomponent versus domino reactions: One-pot free-radical synthesis of β-amino-ethers and β-amino-alcohols
Source: Beilstein J Org Chem. 2015 Jan 15;11:66–73. doi: 10.3762/bjoc.11.10 (PMC4311587; doi:10.3762/bjoc.11.10)
Supplement: File 1 — Analytical data and 1H and 13C NMR spectra of compounds 1a–p. [file Beilstein_J_Org_Chem-11-66-s001.pdf]

Supporting Information

for

Multicomponent versus domino reactions: One-pot free-radical synthesis of β-amino-ethers and β-amino-alcohols

Bianca Rossi, Nadia Pastori[*,§], Simona Prosperini, and Carlo Punta[*,$]

Address: Department of Chemistry, Materials, and Chemical Engineering "G. Natta" - Politecnico di Milano, Via Leonardo da Vinci 32, I-20131 Milano, Italy; Fax (+39)0223993180

Email: Nadia Pastori - nadia.pastori@polimi.it; Carlo Punta - carlo.punta@polimi.it

[*]Corresponding author

[§]phone (+39)0223993012

[$]phone (+39)0223993026

Analytical data and ^{1}H and ^{13}C NMR spectra of compounds **1a–p**

Contents:

Analytical data for compounds **1a–1p**: S2

^{1}H NMR and ^{13}C NMR spectral data for all new compounds: pages S6

4-Methyl-*N*-(2-(tetrahydrofuran-2-yl)propan-2-yl)aniline (1a): Purification by FCC (Hex:AcOEt 7:3) afforded a yellow oil (63% isolated yield). FTIR (liquid film) νmax 3390, 2980, 2881, 1615, 15133, 1110, 811 cm^{-1}; ^{1}H NMR $CDCl_3$ δ (ppm): 1.24-1.25 (m, 6H, $2CH_3$); 1.83-1.95 (m, 4H, $2CH_2$, Cyc), 2.28 (s, 3H, $1CH_3$), 3.62 (sbr, 1H, NH), 3.78-3.85 (m, 2H, $1CH_2$, Cyc), 3.89-3.95 (m, 1H, 1CH, Cyc), 6.78-6.80 (m, 2H, 2CH, Ar), 6.98-7.00 (m, 2H, 2CH, Ar); ^{13}C $CDCl_3$ δ (ppm): 20.6, 23.7, 24.8, 26.4, 26.5, 56.7, 68.7, 86.1, 120.7, 129.2, 129.4, 144.2; GC/MS: 148 (100), 219 (3.3), 106 (6.7). HMRS calcd for $C_{14}H_{21}NO$: 219.1623; found 219.1627.

4-Methyl-*N*-(1-(tetrahydrofuran-2-yl)cyclohexyl)aniline (1b): Purification by FCC (Hex:CH_2Cl_2 5:5) afforded a brown oil (64% isolated yield). FTIR (liquid film) νmax 2929, 2856, 1615, 1513, 1064, 811 cm^{-1}; ^{1}H NMR $CDCl_3$ δ (ppm):): 1.26-1.32 (m, 1H, 1CH, Cyc); 1.47-1.63 (m, 7 H, 7CH, Cyc), 1.76-1.91 (m, 6H, 6CH, Cyc), 2.24 (s, 3H, $1CH_3$), 3.73-3.84 (m, 2H, 1CH, Cyc), 3.97 (m, 1H, 1CH, Cyc), 6.77-6.79 (m, 2H, 2CH, Ar), 6.94-6.96 (m, 2H, 2CH, Ar).; ^{13}C $CDCl_3$ δ (ppm): 20.4, 21.4, 21.5, 25.8, 26.0, 26.3, 31.1, 32.8, 58.4, 68.2, 84.2, 119.0, 127.9, 129.3, 144.1; ESI-MS *m/z* 260 [M^+ + H], 282 [M^+ + Na]; HMRS calcd for $C_{17}H_{25}NO$: 259.1936; found 259.1940

4-Methyl-*N*-(2-(tetrahydrofuran-2-yl)pentan-2-yl)aniline (mixture 1:1 diastereoisomer A and B) (1c): Purification by FCC (Hex:AcOEt 6:4) afforded a yellow oil (57% isolated yield). FTIR (liquid film) νmax 3382, 2958, 2870, 1513, 1067, 809 cm^{-1}; ^{1}H NMR $CDCl_3$ δ (ppm): 0.91 (t, 3H, 1 CH_3); 0.93 (t, 6H, 2 CH_3); 1.20 (s, 3H, 1 CH_3), 1.37-1.51 (m, 4H, $2CH_2$), 1.58-1.65 (m, 3H, $1CH+1CH_2$), 1.69-1,77 (m, 1H, CH), 1.80-1.93 (m, 8H, $4CH_2$, Cyc), 2.26 (s, 6H, $2CH_3$), 3.75-3.83 (m, 2H, 2CH , Cyc), 3.86-3.94 (m, 4H, 4CH, Cyc), 6.72-6.74 (2m, 4H, 4CH, Ar), 6.96-6.98 (m, 4H, 4CH, Ar); ^{13}C $CDCl_3$ δ (ppm): 14.8, 14.9, 17.1, 17.3, 20.6, 21.8, 22.0, 26.3, 26.3, 26.4, 26.5, 40.0, 40.3, 58.9, 59.0, 68.5, 68.7, 84.3, 84.9, 119.2, 119.5, 128.2, 128.5, 129.5, 144.3, 144.75; GC/MS: 176 (100), 247 (3.3), 106 (6.7). HMRS calcd for $C_{16}H_{25}NO$: 247.1936; found 247.1934

***N*-(4-(*tert*-Butyl)-1-(tetrahydrofuran-2-yl)cyclohexyl)-4-methylaniline (1d):** Purification by FCC (Hex:CH_2Cl_2 5:5) afforded a white powder (49% isolated yield), mp 91-93°C. FTIR (liquid film) νmax 3410, 2943, 2863, 1615, 1448, 1069, 810 cm^{-1}; ^{1}H NMR $CDCl_3$ δ (ppm): 0.84 (s, 9H, tBu); 1.02 (dt, 1H, CH); 1.18-1.29 (m, 1H, CH); 1.31-1.42 (m, 2H, 2CH); 1.46-1.61 (m, 3H, 3CH); 1.74-1.89 (m, 5H, 5CH); 2.07-2.12 (m, 1H, 1CH); 2.24 (s, 3H, CH_3), 3.72-3.81 (m, 2H, 2CH, Cyc), 3.90 (m, 1H, 1CH, Cyc), 6.77-6.79 (m, 2H, 2CH, Ar), 6.94-6.96 (m, 2H, 2CH, Ar); ^{13}C $CDCl_3$ δ (ppm): 20.6, 22.0, 22.1, 26.2, 26.4, 27.7, 31.0, 32.6, 32.9, 48.1, 58.3, 68.4, 85.2, 118.4, 127.7, 129.5, 144.3; ESI-MS *m/z* 316 [M^+ + H], 338 [M^+ + Na]; HMRS calcd for $C_{21}H_{33}NO$ 315.2562; found 315.2560

4-Methyl-*N*-(4-phenyl-1-(tetrahydrofuran-2-yl)cyclohexyl)aniline (1e): Purification by FCC (Hex:CH_2Cl_2 6:4) afforded a white powder (40% isolated yield), mp 104-106°C. FTIR (liquid film) νmax 2923, 2858, 1614, 1513, 1255, 757, 699 cm^{-1}; ^{1}H NMR $CDCl_3$ δ (ppm): 1.52-1.60 (m, 1H, 1CH, Cyc); 1.65-1.91 (m, 9H, 9 CH); 1.93-1.98 (m, 1 H, 1CH, Cyc), 2.08-2.14 (m, 1H, 1CH, Cyc), 2.22 (s, 3H, $1CH_3$), 2.52

(m, 1H, 1CH, Cyc), 3.70-3.81 (m, 2H, $1CH_2$, Cyc), 3.91 (m, 1H, 1CH., Cyc); 6.78-6.80 (m, 2H, 2CH, Ar), 6.93-6.95 (m, 2H, 2CH, Ar), 7.11-7.16 (m, 1H, CH Ar); 7.18-7.26 (m, 5H, 4CH, Ar); ^{13}C CDCl3 δ (ppm): 20.6, 26.3, 26.4, 28.9, 29.0, 31.2, 32.8, 44.4, 58.4, 68.4, 84.9, 118.9, 126.1 127.0, 128.3, 128.4, 129.6, 144.2, 147.3; ESI-MS *m/z* 336 [M^+ + H], 358 [M^+ + Na]; HMRS calcd for $C_{23}H_{29}NO$: 335.2249; found 335.2253.

4-Methoxy-*N*-(2-(tetrahydrofuran-2-yl)propan-2-yl)aniline (1f): Purification by FCC (Hex:CH_2Cl_2 7:3) afforded a yellow oil (57% isolated yield). FTIR (liquid film) νmax 2972, 2868, 1509, 1463, 1241, 1068,1038, 527 cm^{-1}; ^{1}H NMR $CDCl_3$ δ (ppm): 1.14 (s, 6H, $2CH_3$); 1.79-1.93 (m, 4 H, $2CH_2$, Cyc), 3.71-3.76 (m, 1H, CH, Cyc), 3.74 (s, 3H, OCH_3), 3.76-3.81 (m, 1H, CH, Cyc), 3.85-3.91 (m,1H, CH, Cyc); 6.73-6.75 (m, 2H, 2CH, Ar), 6.83-6.85 (m, 2H, 2CH, Ar); ^{13}C CDCl3 δ (ppm): 23.4, 24.8, 26.4, 26.6, 55.6, 57.1, 68.7, 85.8, 114.2, 123.9, 139.5, 154.8; ESI-MS m/z 236 [M^+ + H], 258 [M^+ + Na]; HMRS calcd for $C_{14}H_{21}NO_2$: 235.1572; found 235.1575.

4-Chloro-*N*-(2-(tetrahydrofuran-2-yl)propan-2-yl)aniline (1g): Purification by FCC (Hex:AcOEt 8:2) afforded a light brown powder (69% isolated yield), mp 48-50°C. FTIR (liquid film) νmax 3381, 2973, 2869, 1597, 1491, 1069, 817 cm^1; ^{1}H NMR $CDCl_3$ δ (ppm): 1.24 (s, 3H, CH_3); 1.26 (s, 3H, CH_3); 1.77-.1.93 (m, 4 H, $2CH_2$, Cyc), 3.74-3.82 (m, 2H, 2CH, Cyc), 3.87-3.92 (m, 1H, 1CH, Cyc), 6.74-6.76 (m, 2H, 2CH, Ar), 7.08-7.11 (m, 2H, 2CH, Ar); ^{13}C $CDCl_3$ δ (ppm): 23.5, 24.7, 26.3, 26.5, 56.7, 68.8, 86.1, 120.4, 124.2, 128.8, 145.5; ESI-MS m/z 240 [M^+ + H], 262 [M^+ + Na]; HMRS calcd for $C_{13}H_{18}ClNO$ (^{35}Cl): 239.1077; found 239.1077.

***N*-(2-(1,3-Dioxolan-2-yl)propan-2-yl)-4-methylaniline (1h)**: Purification by FCC (Hex:AcOEt 7:3) afforded a yellow oil (18% isolated yield). FTIR (liquid film) νmax 3393, 2977, 2879, 1615, 1513, 1111, 810 cm^{-1}; ^{1}H NMR $CDCl_3$ δ (ppm): 1.25 (s, 6H, $2CH_3$); 2.26 (s, 3H, CH_3), 3.89- 4.02 (m, 4H, $2CH_2$, Cyc), 4.75 (s, 1H, 1CH, Cyc), 6.79-6.81 (m, 2H, 2CH, Ar), 6.97-6.99 (m, 2H, 2CH, Ar); ^{13}C $CDCl_3$ δ (ppm): 20.7, 22.9, 57.1, 65.7, 108.0, 113.3, 121.4, 129.4, 130.1, 143.4; GC/MS: 148 (100), 221 (2.8), 106 (5.7). HMRS calcd for $C_{13}H_{19}NO_{2:}$ 221.1416; found 221.1413

***N*-(2-(1,4-Dioxan-2-yl)propan-2-yl)-4-methylaniline (1i):** Purification by FCC (Hex:AcOEt 75:25) afforded a yellow oil (30% isolated yield). FTIR (liquid film) νmax 3379, 2972, 2855, 1513, 1121, 908 cm^{-1}; ^{1}H NMR $CDCl_3$ δ (ppm): 1.20-1.21 (m, 6H, $2CH_3$), 2.27 (s, 3H, $1CH_3$), 3.46-3.60 (m, 3H, $1CH_2$, 1CH, Cyc), 3.66-3.75 (m, 2H, $1CH_2$, Cyc), 3.84-3.87 (m, 2H, $1CH_2$, Cyc), 6.76-6.78 (m, 2H, 2CH, Ar), 6.98-7.01 (m, 2H, 2CH, Ar); ^{13}C $CDCl_3$ δ (ppm): 20.7, 24.0, 24.6, 55.9, 66.5, 67.4, 67.6, 80.6, 121.5, 129.4, 130.1, 143.3; GC/MS: 148 (100), 235 (2.3), 106 (6.7). HMRS calcd for $C_{14}H_{21}NO_2$ 235.1572; found 235.1582.

***N*-(3-Ethoxy-2-methylbutan-2-yl)-4-methylaniline (1j)**: Purification by FCC (Hex:AcOEt 9:1) afforded a yellow oil (25% isolated yield). FTIR (liquid film) νmax 3385, 2975, 2870, 1513, 1111, 816 cm^{-1}; ^{1}H NMR $CDCl_3$ δ (ppm): 1.13-1.14 (d, 3H, $1CH_{3,}$ J=6.2 Hz); 1.19 (s, 3H, $1CH_3$), 1.22 (t, 3H, $CH_{3,}$ J=6.9 Hz), 1.23 (s, 3H, $1CH_3$), 2.26 (s, 3H, $1CH_3$), 3.31 (dd, 1H, 2CH , J=6.2 Hz), 3.35 (tt, 1H, $1CH_2$, J=6.9 Hz), 3.64 (tt, 1H,

1CH$_2$, J=6.9 Hz), 6.74-6.76 (m, 2H, 2CH, Ar), 6.96-6.99 (m, 2H, 2CH, Ar); ^{13}C CDCl$_3$ δ (ppm): 14.0, 15.8, 20.6, 23.0, 25.0, 57.9, 64.9, 80.8, 120.5, 129.1, 129.4, 144.3; GC/MS: 148 (100), 221 (0.9), 106 (4.5). HMRS calcd for $C_{14}H_{23}NO$: 221.1780; found 221.1792.

2-Methyl-2-(*p*-tolylamino)propa-1-ol (1k): characterization is reported in literature [27]

3-Methyl-3-(*p*-tolylamino)butan-2-ol (1l): Purification by FCC (Hex:AcOEt 7:3) afforded a pail liquid (64% isolated yield). FTIR (liquid film) νmax 1903, 1611, 1436, 1041, 831, 540 cm^{-1}; ^{1}H NMR CDCl$_3$ δ (ppm): 1.13 (s, 3H, CH$_3$); 1.16 (s, 3H, CH$_3$); 1.19 (d, 3H, 1CH$_3$, J=6.5Hz), 2.27 (s, 3H, 1CH$_3$), 3.80-3.85 (q, 1H, 1CH, J= 6.5Hz), 6.76-6.78 (m, 2H, 2CH, Ar), 6.99-7.01 (m, 2H, 2CH, Ar); ^{13}C CDCl3 δ (ppm): 17.0, 20.5, 22.0, 23.7, 58.9, 72.1, 121.5, 129.4, 130.8, 142.4; GC/MS: 148 (100), 193 (3), 106 (6); HMRS calcd for $C_{12}H_{19}NO$ 193.1467; found 193.1468.

1-(1-(*p*-Tolylamino)cyclohexyl)ethanol (1m): Purification by FCC (Hex:AcOEt 9:1) afforded a pale liquid (75% isolated yield). FTIR (liquid film) νmax 3407, 2931, 2858, 1615, 1514, 1247, 809 cm^{-1}; ^{1}H NMR DMSO δ (ppm): 1.01 (d, 3H, 1CH$_3$, J=6.4Hz), 1.13-1.18 (m, 1H, CH Cyc), 1.41-1.55 (m, 7H, 5CH Cyc); 1.70-1.73 (m, 1H, CH Cyc); 1.86-1.89 (m, 1H, CH Cyc); 2.13 (s, 3H, 1CH$_3$), 3.82 (q, 1H, 1CH, J= 6.4Hz), 4.28 (sbr, 1H, NH), 4.33 (sbr, 1H, OH); 6.68-6.70 (m, 2H, 2CH, Ar), 6.82-6.84 (m, 2H, 2CH, Ar); ^{13}C DMSO δ (ppm): 17.2, 19.8, 20.8, 20.9, 25.6, 28.9, 30.3, 58.6, 69.7, 116.3, 124.5, 128.7, 144.8; GC/MS: 188 (100), 233 (2), 106 (8); HMRS calcd for $C_{15}H_{23}NO$: 233.1780; found 233.1780.

3-Methyl-3-(*p*-tolylamino)hexan-2-ol (**1n**): two isomers mixture 50/50; purification by FCC (Hex:AcOEt 8:2) afforded an oil (41% isolated yield). FTIR (liquid film) νmax 3378, 2958, 2871, 1615, 1514, 1455, 1247, 1111, 812 cm^{-1}; ^{1}H NMR CDCl$_3$ δ (ppm): 0.83 (t, 3H, CH$_3$); 0.96 (t, 3H, CH$_3$), 1.10 (s, 3H, CH$_3$), 1.11 (s, 3H, CH$_3$), 1.19 (d, 3H, CH$_3$), 1.20 (d, 3H, CH$_3$), 1.26-1.70 (m, 8H, 4CH$_2$), 2.26 (s, 6H, 2 CH$_3$), 3.90-3.95 (m, 2H, 2CH), 6.70-6.75 (m, 4H, 4CH, Ar), 6.97-7.00 (m, 4H, 4CH, Ar); ^{13}C CDCl$_3$ δ (ppm): 14.5, 14.8, 16.4, 16.7, 16.9, 17.0, 20.1, 20.4, 20.4, 21.3, 3.75, 3.80, 60.4, 61.2, 70.8, 72.2, 119.3, 120.2, 129.3, 129.5, 129.5, 129.8, 143.2, 143.3; GC/MS: 221 (3), 176 (100),160 (4), 146 (8), 134 (2), 106 (8). HMRS calcd for $C_{14}H_{23}NO$: 221.17797; found 221.17811.

2-Methyl-2-(*p*-tolylamino)pentan-3-ol (1o): Purification by FCC (Hex:AcOEt 9:1) afforded white crystals (61% isolated yield). FTIR (liquid film) vmax 3296, 2963, 2929, 2359, 1513, 1192, 830 cm-1; ^{1}H NMR CDCl$_3$ δ (ppm): 1.08 (t, 3H, 1CH$_3$), 1.13 (s, 3H, 1CH$_3$), 1.14 (s, 3H, 1CH$_3$), 1.39 (m, 1H, CH), 1.60 (m, 1H, CH); 3.50 (dd, 1H, CH); 6.76-6.78 (m, 2H, 2CH, Ar), 7.00-7.02 (m, 2H, 2CH, Ar); ^{13}C CDCl$_3$ δ (ppm): 11.9, 20.6, 22.8, 23.9, 24.3, 58.7, 78.1, 121.4, 129.5, 130.7, 142.6; GC/MS: 148 (100), 207 (1.3), 106 (6.7); HMRS calcd for $C_{13}H_{21}NO$: 207.1623; found 207.1645.

1-(1-(*p*-Tolylamino)cyclohexyl)ethanol (1p): Purification by FCC (Hex:AcOEt 9:1) afforded a pail liquid (60% isolated yield). FTIR (liquid film) νmax 3407, 2931, 2858, 1615, 1514, 1247, 809 cm^{-1}; ^{1}H NMR DMSO δ (ppm): 1.01 (d, 3H, 1CH$_3$, J=6.4Hz), 1.13-1.18 (m, 1H, CH Cyc), 1.41-1.55 (m, 7H, 5CH Cyc);

1.70-1.73 (m, 1H, CH Cyc); 1.86-1.89 (m, 1H, CH Cyc); 2.13 (s, 3H, 1CH_3), 3.82 (q, 1H, 1CH, J= 6.4Hz), 4.28 (sbr, 1H, NH), 4.33 (sbr, 1H, OH); 6.68-6.70 (m, 2H, 2CH, Ar), 6.82-6.84 (m, 2H, 2CH, Ar); ^{13}C DMSO δ (ppm): 17.2, 19.8, 20.8, 20.9, 25.6, 28.9, 30.3, 58.6, 69.7, 116.3, 124.5, 128.7, 144.8; GC/MS: 188 (100), 233 (2), 106 (8); HMRS calcd for $C_{15}H_{23}NO$: 233.1780; found 233.1780.

4-(Tetrahydrofuran-2-yl)-4-(*p*-tolylamino)butan-1-ol (2a): characterization is reported in literature [30].

3-(*p*-Tolylamino)butan-2-ol (2b): characterization is reported in literature [29].

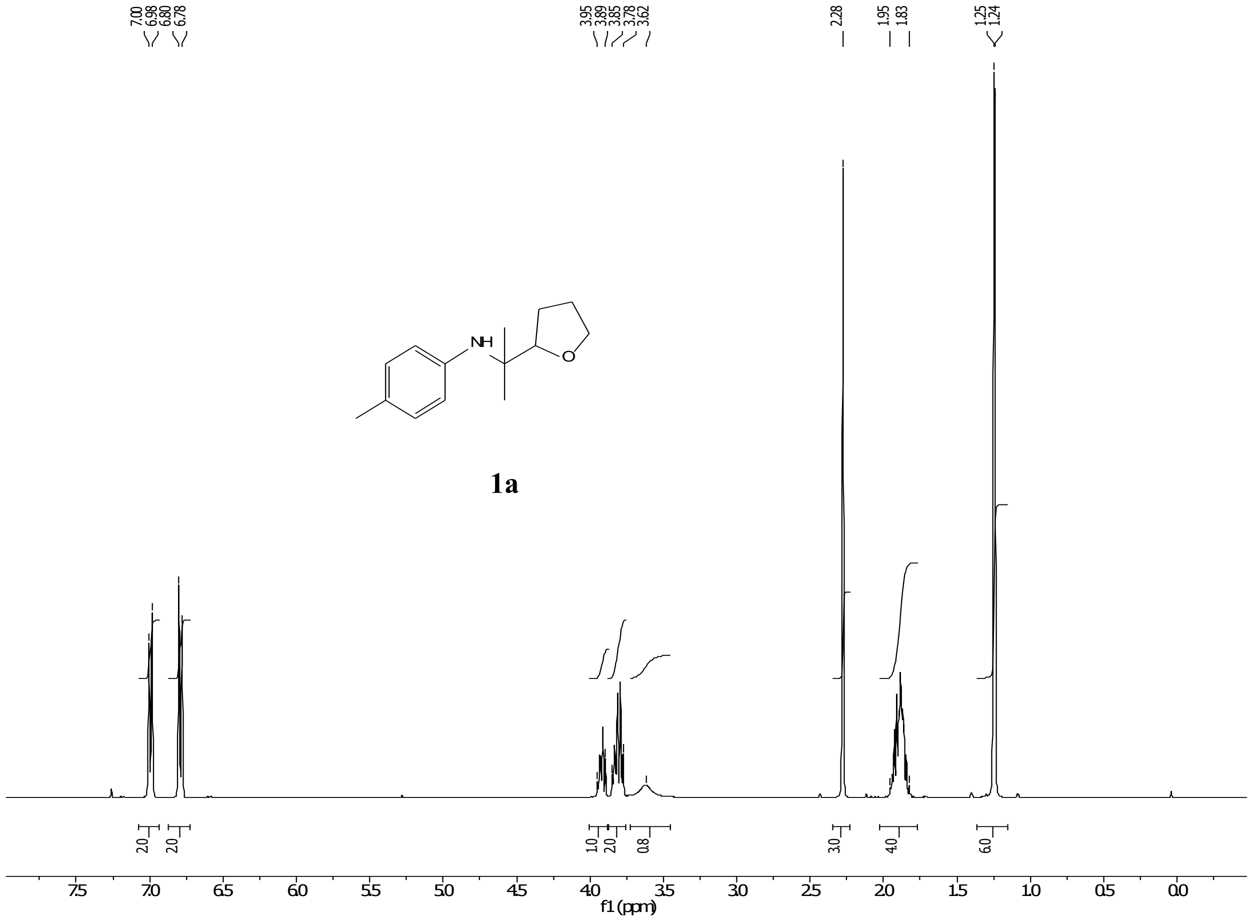
7.00
6.98
6.80
6.78
3.95
3.89
3.85
3.78
3.62
2.28
1.95
1.83
1.25
1.24
NH
O
1a
2.0
2.0
1.0
2.0
0.8
3.0
4.0
6.0
7.5 7.0 6.5 6.0 5.5 5.0 4.5 4.0 3.5 3.0 2.5 2.0 1.5 1.0 0.5 0.0
f1 (ppm)

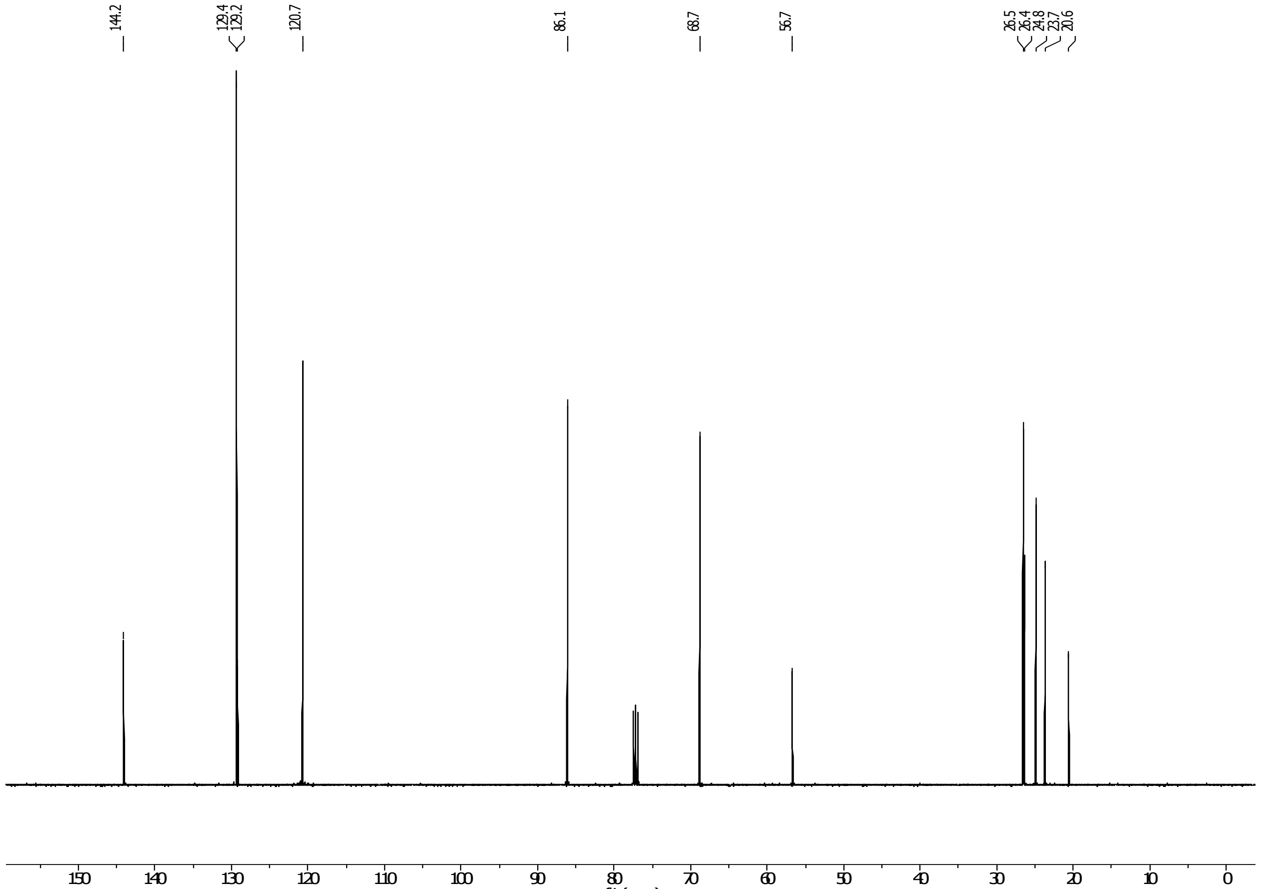
144.2
129.4
129.2
120.7
86.1
68.7
56.7
26.5
26.4
24.8
23.7
20.6
150 140 130 120 110 100 90 80 70 60 50 40 30 20 10 0
f1 (ppm)

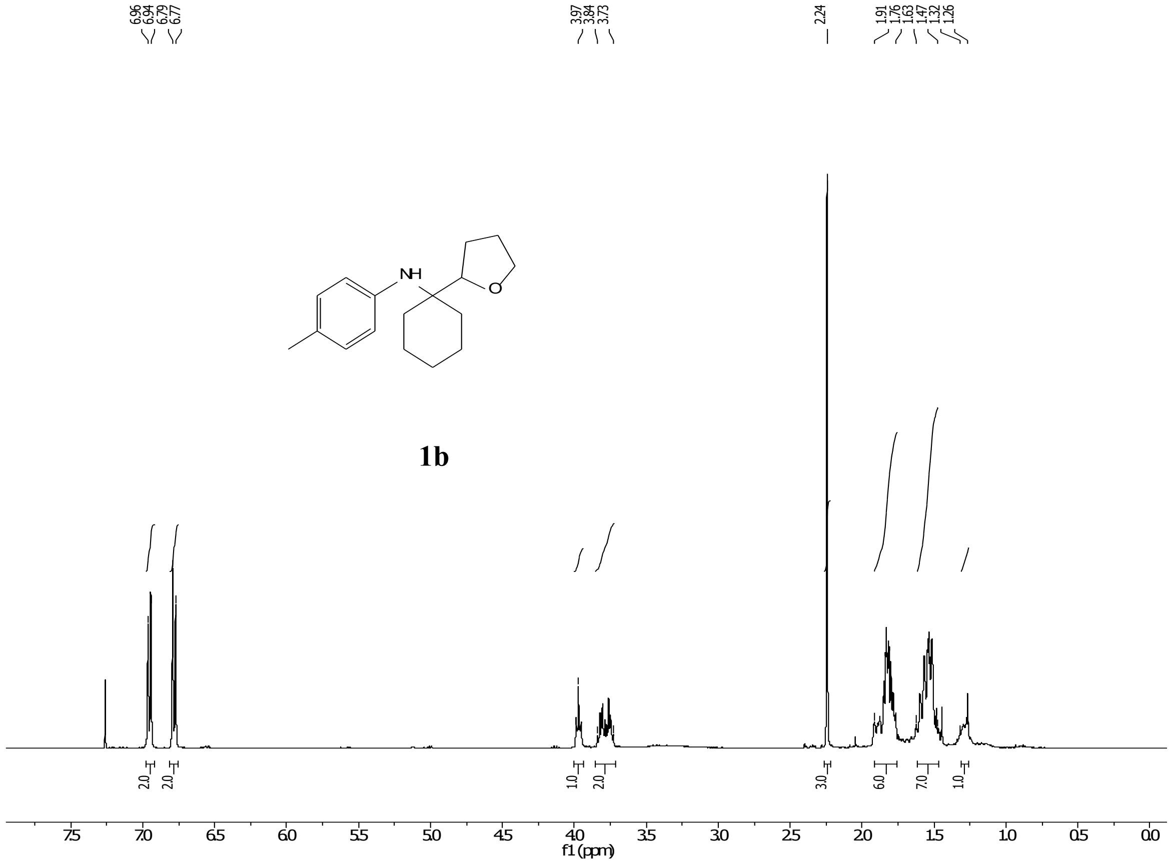
6.96
6.94
6.79
6.77
3.97
3.84
3.73
2.24
1.91
1.76
1.63
1.47
1.32
1.26
NH
O
1b
2.0
2.0
1.0
2.0
3.0
6.0
7.0
1.0
7.5 7.0 6.5 6.0 5.5 5.0 4.5 4.0 3.5 3.0 2.5 2.0 1.5 1.0 0.5 0.0
f1 (ppm)

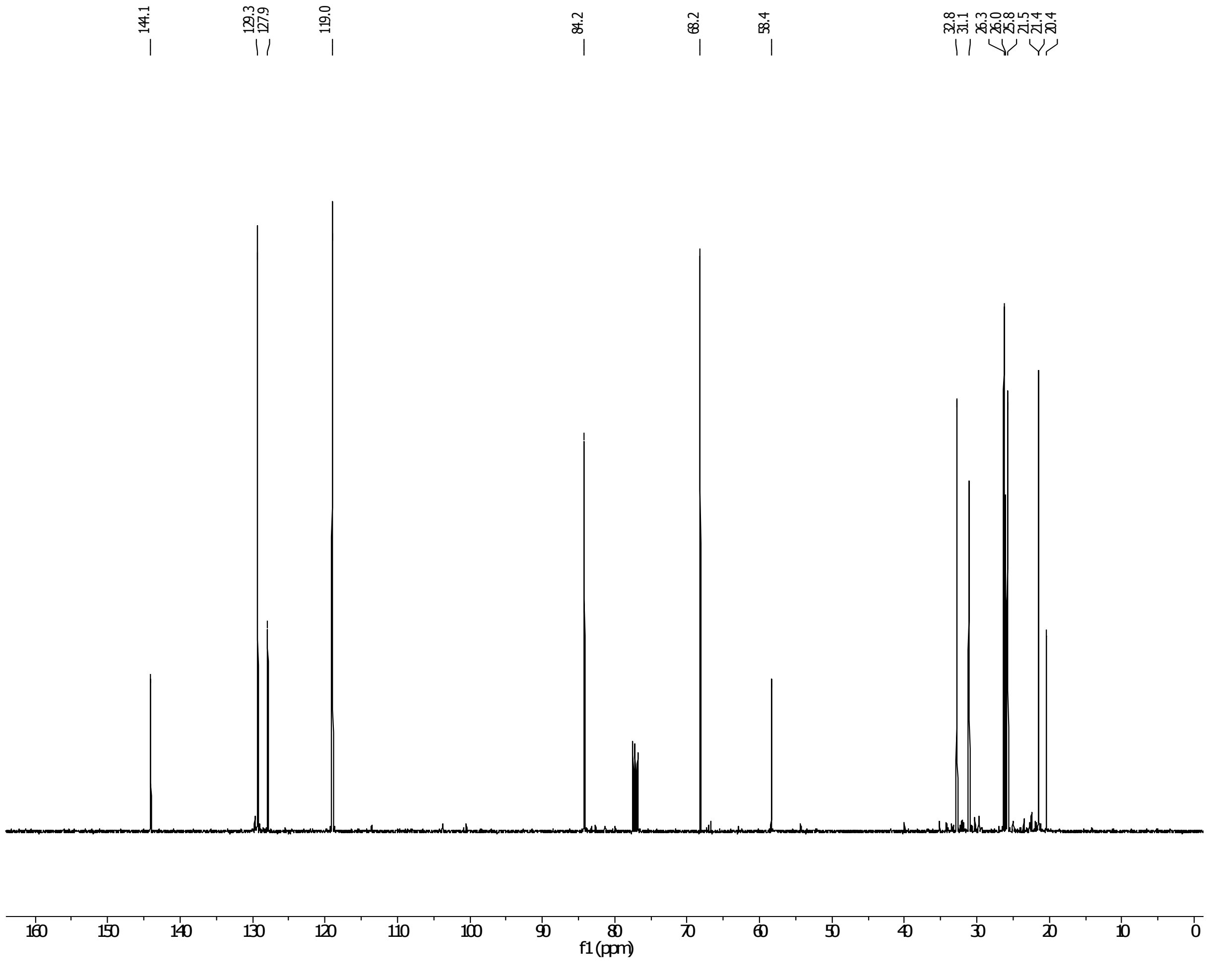
144.1
129.3
127.9
119.0
84.2
68.2
58.4
32.8
31.1
26.3
26.0
25.8
21.5
21.4
20.4
160 150 140 130 120 110 100 90 80 70 60 50 40 30 20 10 0
f1 (ppm)

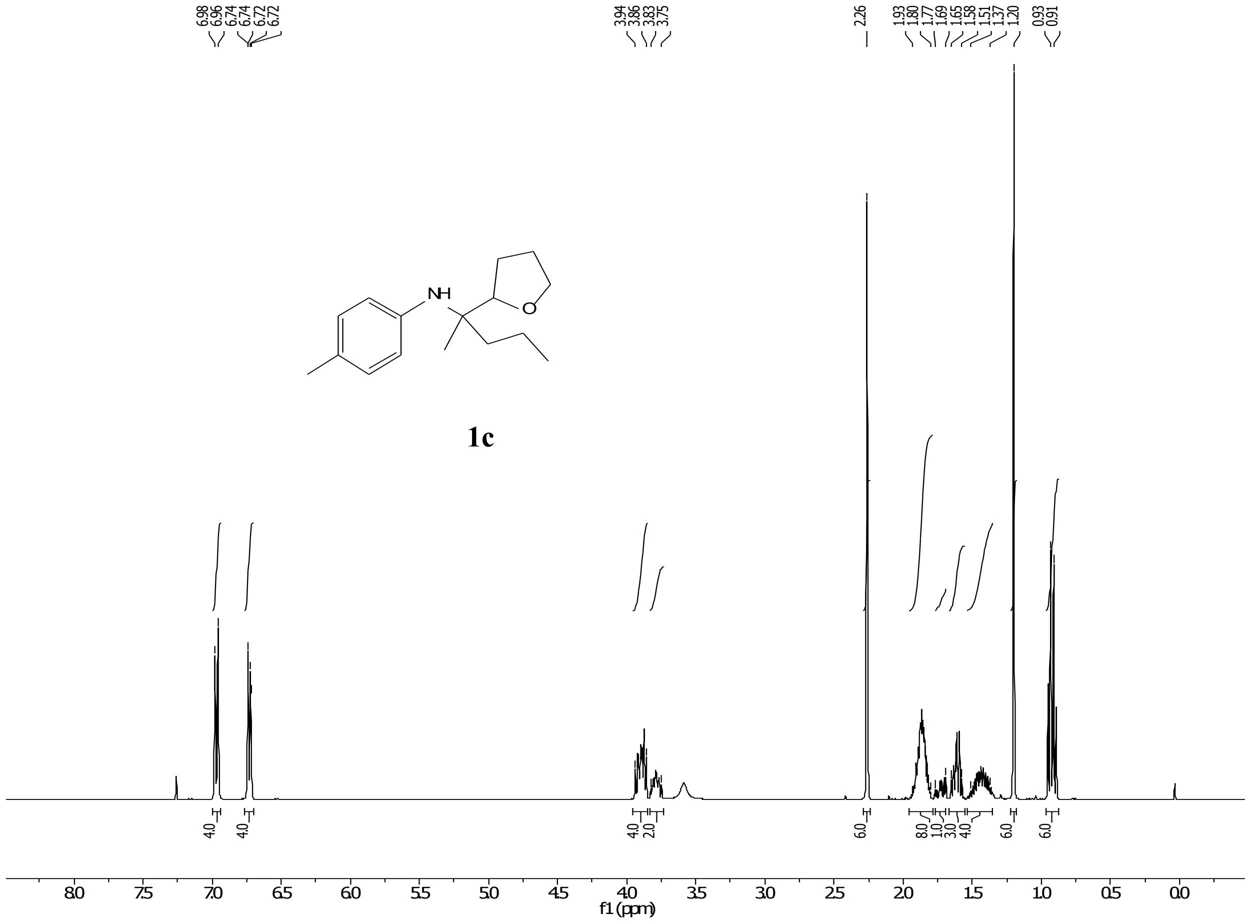
6.98 6.96 6.74 6.74 6.72 6.72
3.94 3.86 3.83 3.75
2.26
1.93 1.80 1.77 1.69 1.65 1.58 1.51 1.37 1.20
0.93 0.91
NH
O
1c
4.0 4.0 4.0 2.0 6.0 8.0 1.0 3.0 4.0 6.0 6.0
8.0 7.5 7.0 6.5 6.0 5.5 5.0 4.5 4.0 3.5 3.0 2.5 2.0 1.5 1.0 0.5 0.0
f1 (ppm)

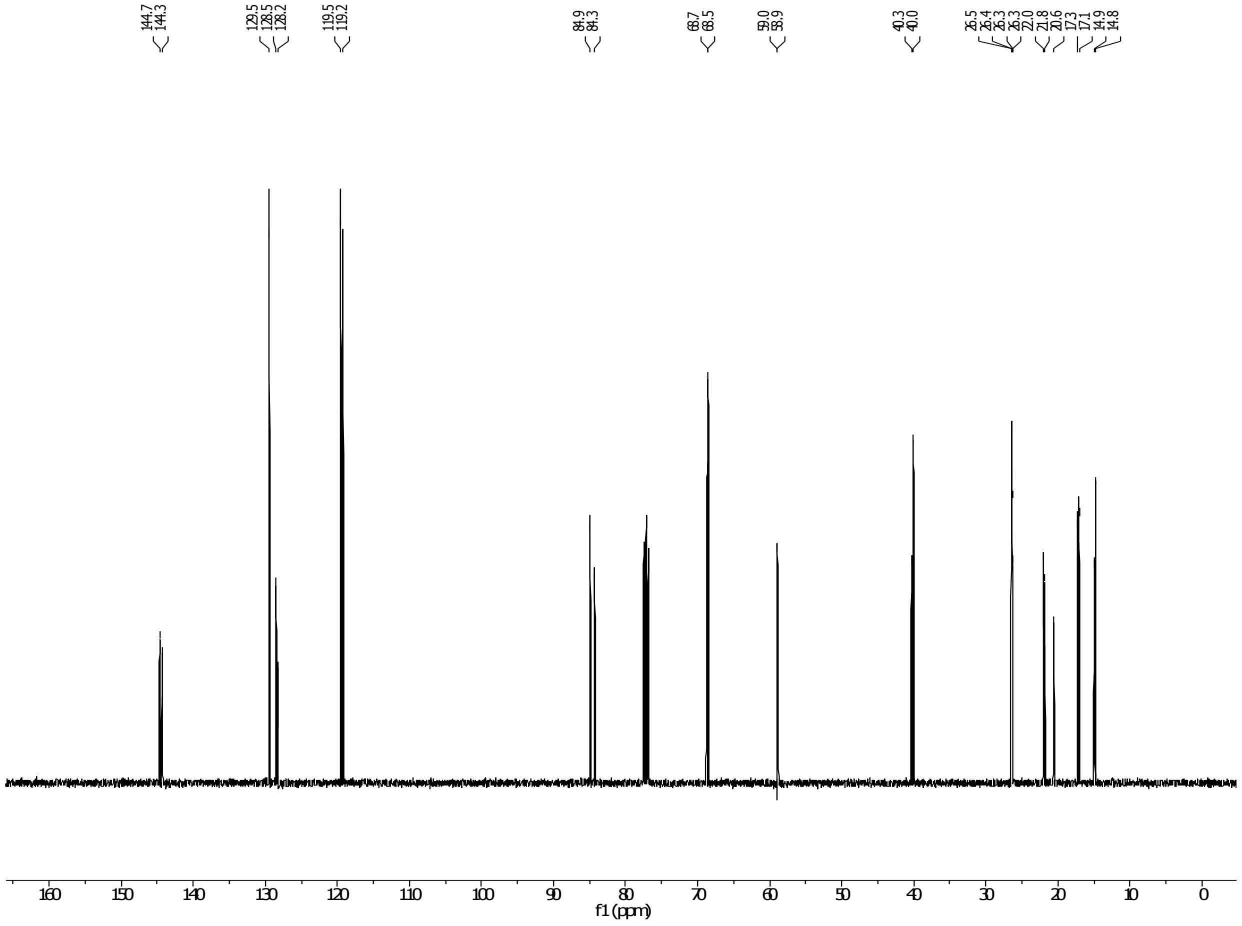
144.7 144.3
129.5 128.5 128.2
119.5 119.2
84.9 84.3
68.7 68.5
59.0 58.9
40.3 40.0
26.5 26.4 26.3 26.3 22.0 21.8 20.6 17.3 17.1 14.9 14.8
160 150 140 130 120 110 100 90 80 70 60 50 40 30 20 10 0
f1 (ppm)

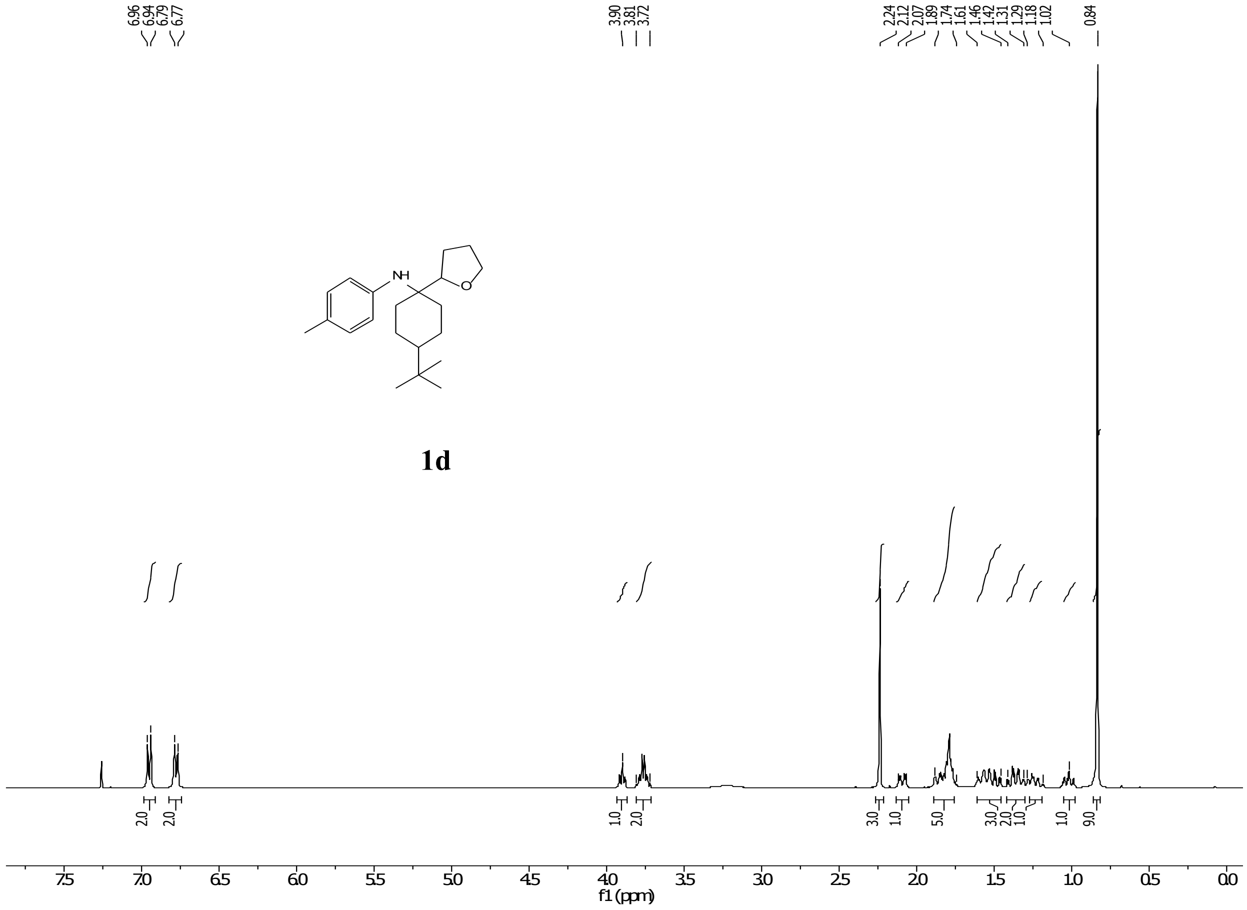
6.96
6.94
6.79
6.77
3.90
3.81
3.72
2.24
2.12
2.07
1.89
1.74
1.61
1.46
1.42
1.31
1.29
1.18
1.02
0.84
NH
O
2.0
2.0
1.0
2.0
3.0
1.0
5.0
3.0
2.0
1.0
1.0
9.0
7.5 7.0 6.5 6.0 5.5 5.0 4.5 4.0 3.5 3.0 2.5 2.0 1.5 1.0 0.5 0.0
f1 (ppm)

1d

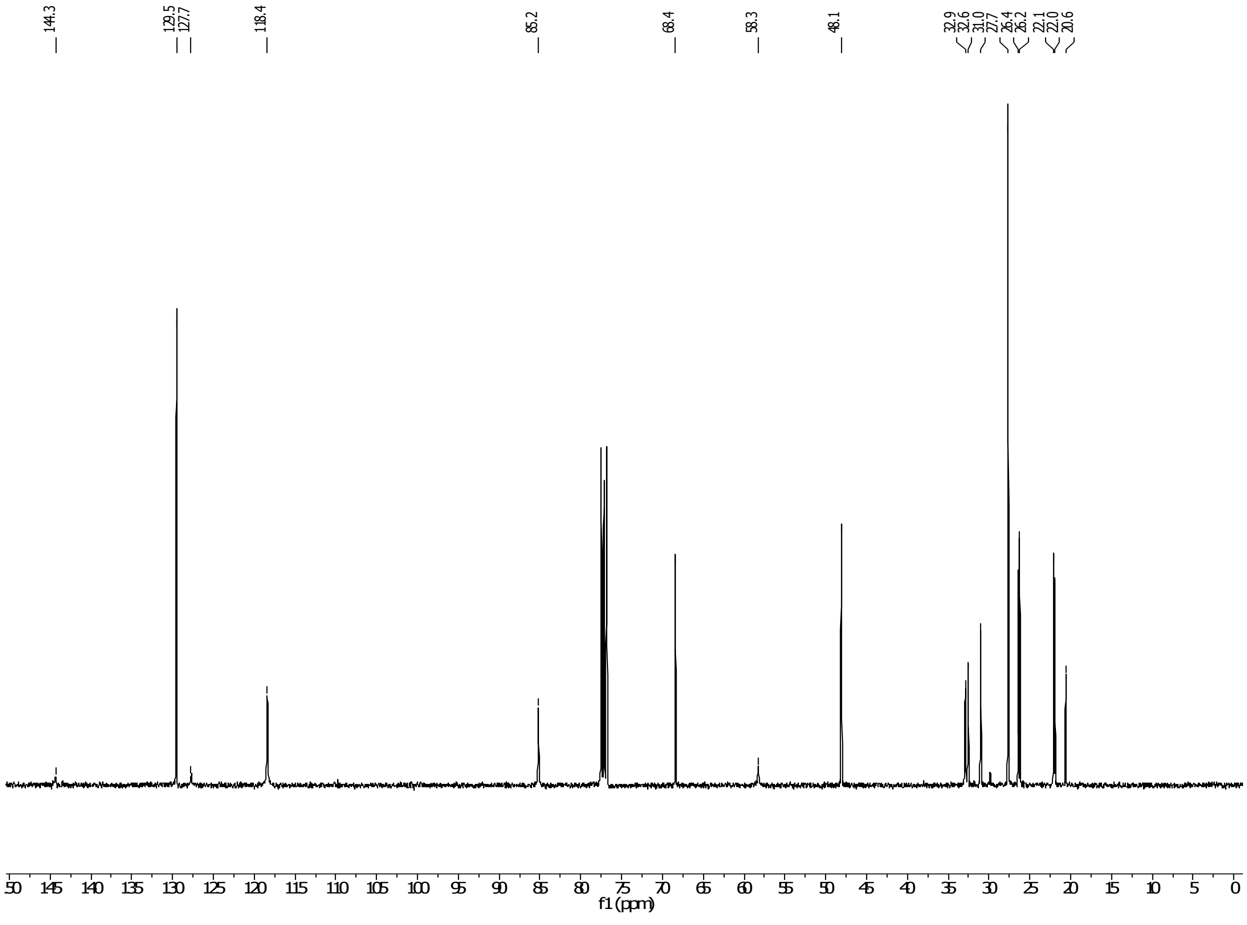
144.3
129.5
127.7
118.4
85.2
68.4
58.3
48.1
32.9
32.6
31.0
27.7
26.4
26.2
22.1
22.0
20.6
150 145 140 135 130 125 120 115 110 105 100 95 90 85 80 75 70 65 60 55 50 45 40 35 30 25 20 15 10 5 0
f1 (ppm)

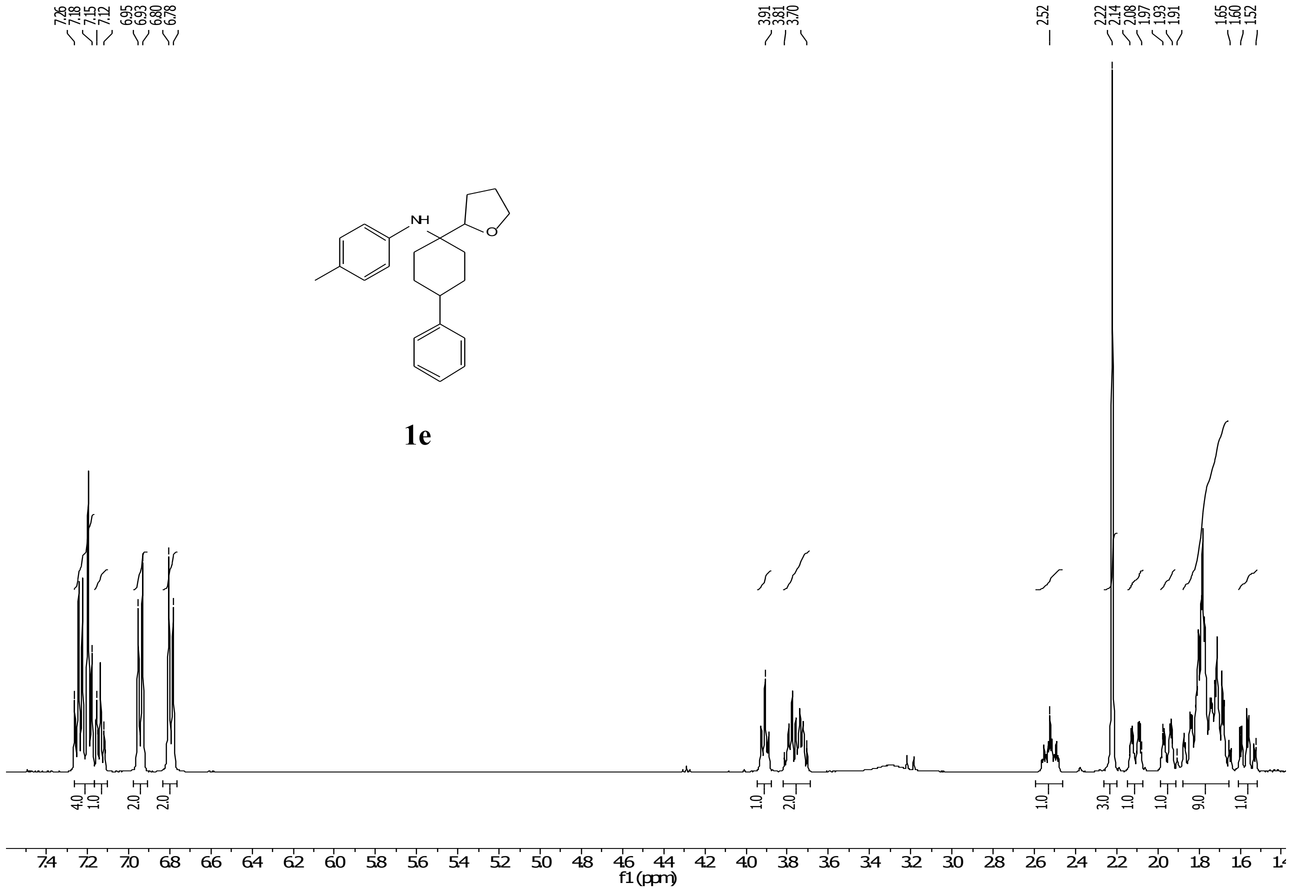
7.26 7.18 7.15 7.12 6.95 6.93 6.80 6.78
3.91 3.81 3.70
2.52
2.22 2.14 2.08 1.97 1.93 1.91
1.65 1.60 1.52
NH
O
1e
4.0 1.0 2.0 2.0 1.0 2.0 1.0 3.0 1.0 1.0 9.0 1.0
f1 (ppm)

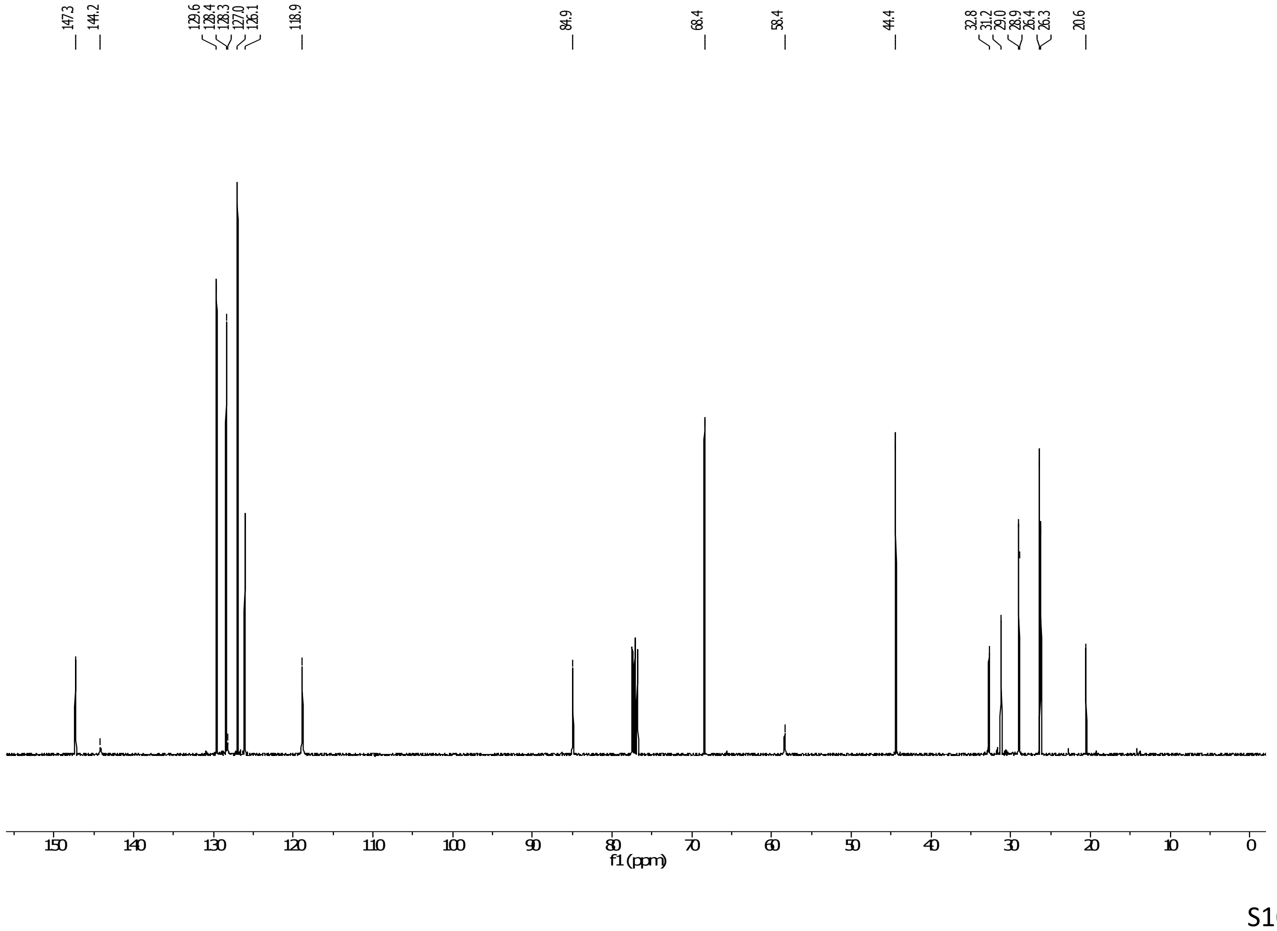
147.3 144.2
129.6 128.4 128.3 127.0 126.1 118.9
84.9
68.4
58.4
44.4
32.8 31.2 29.0 28.9 26.4 26.3 20.6
f1 (ppm)

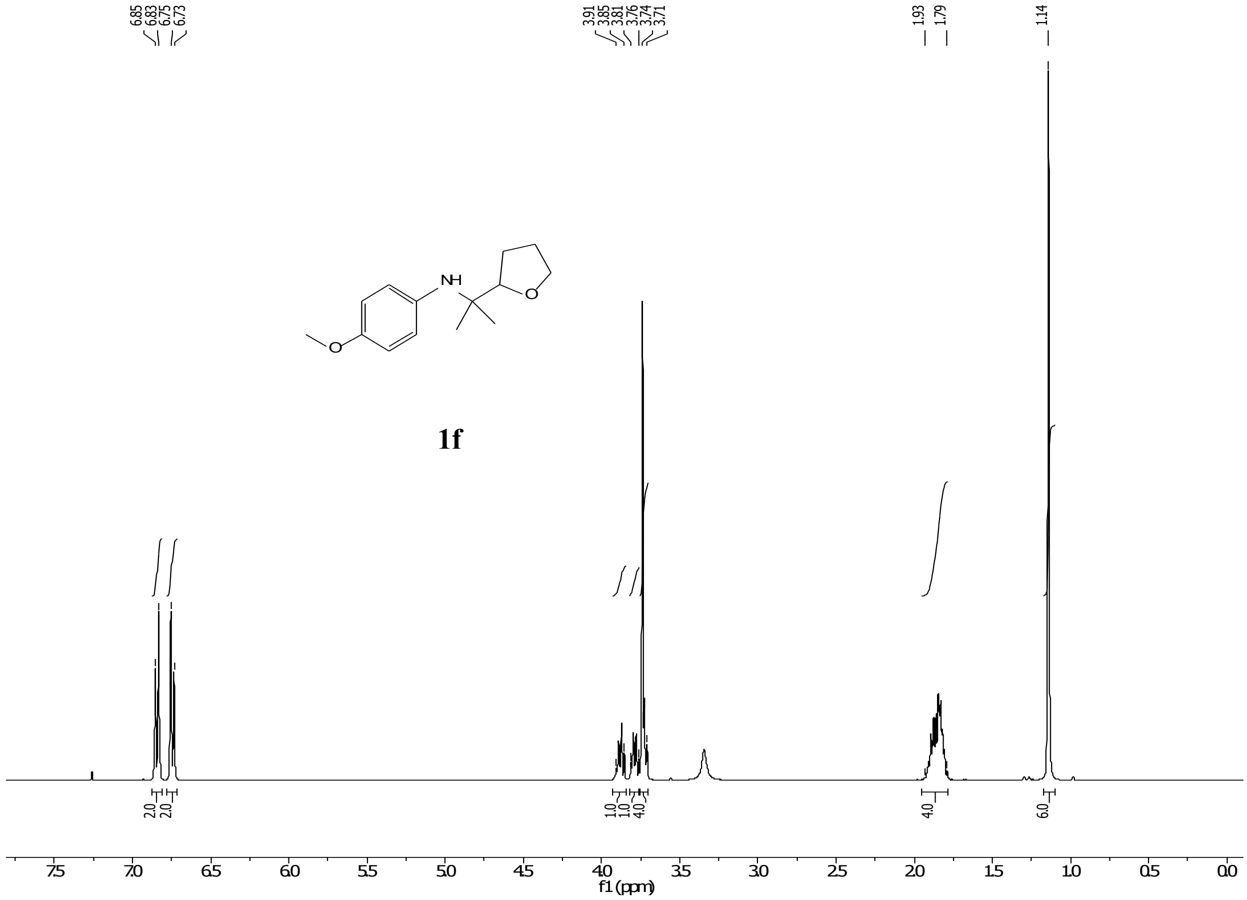
6.85
6.83
6.75
6.73
3.91
3.85
3.81
3.76
3.74
3.71
1.93
1.79
1.14
NH
O
O
1f
2.0
2.0
1.0
1.0
4.0
4.0
6.0
7.5 7.0 6.5 6.0 5.5 5.0 4.5 4.0 3.5 3.0 2.5 2.0 1.5 1.0 0.5 0.0
f1 (ppm)

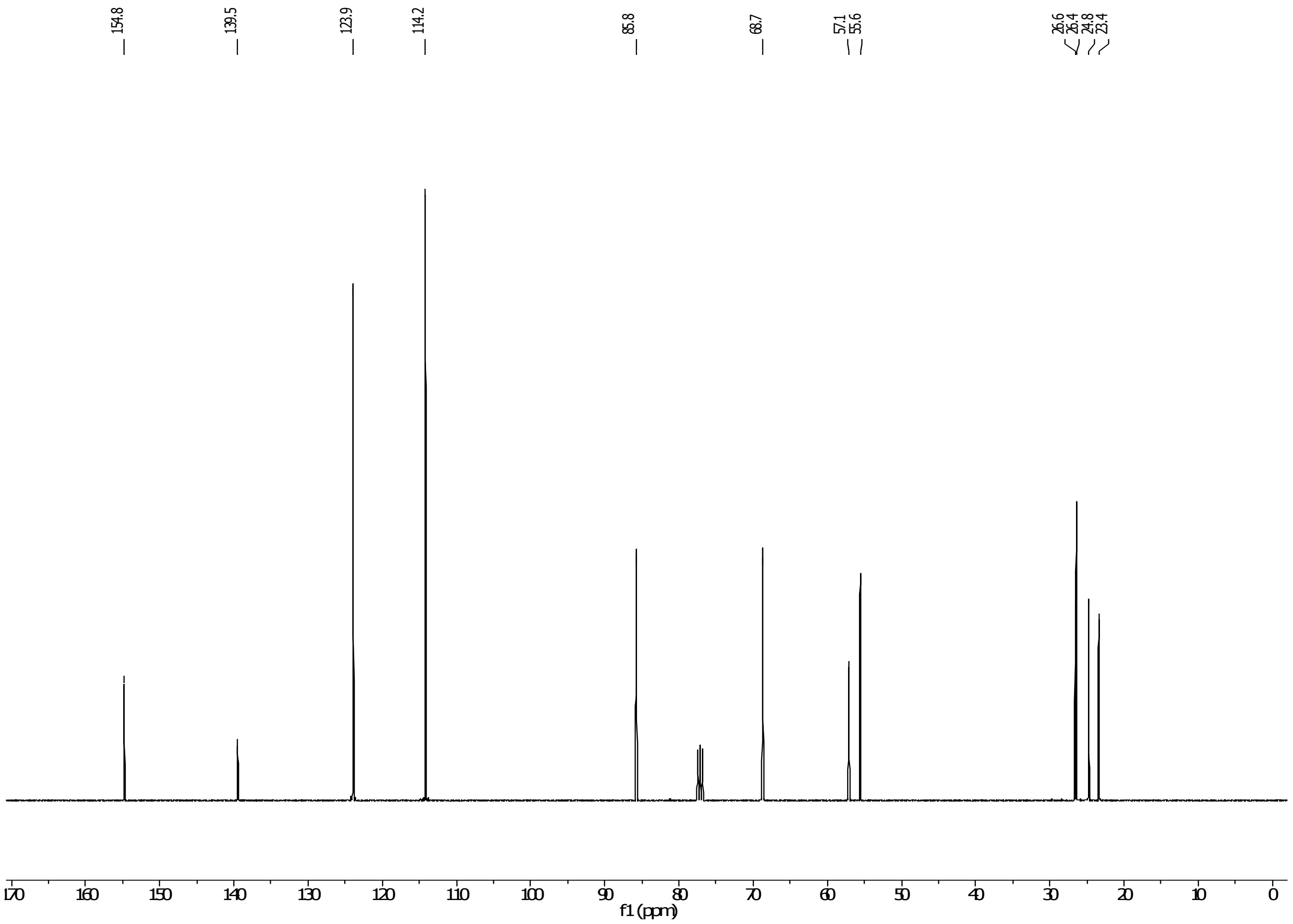
154.8
139.5
123.9
114.2
85.8
68.7
57.1
55.6
26.6
26.4
24.8
23.4
170 160 150 140 130 120 110 100 90 80 70 60 50 40 30 20 10 0
f1 (ppm)

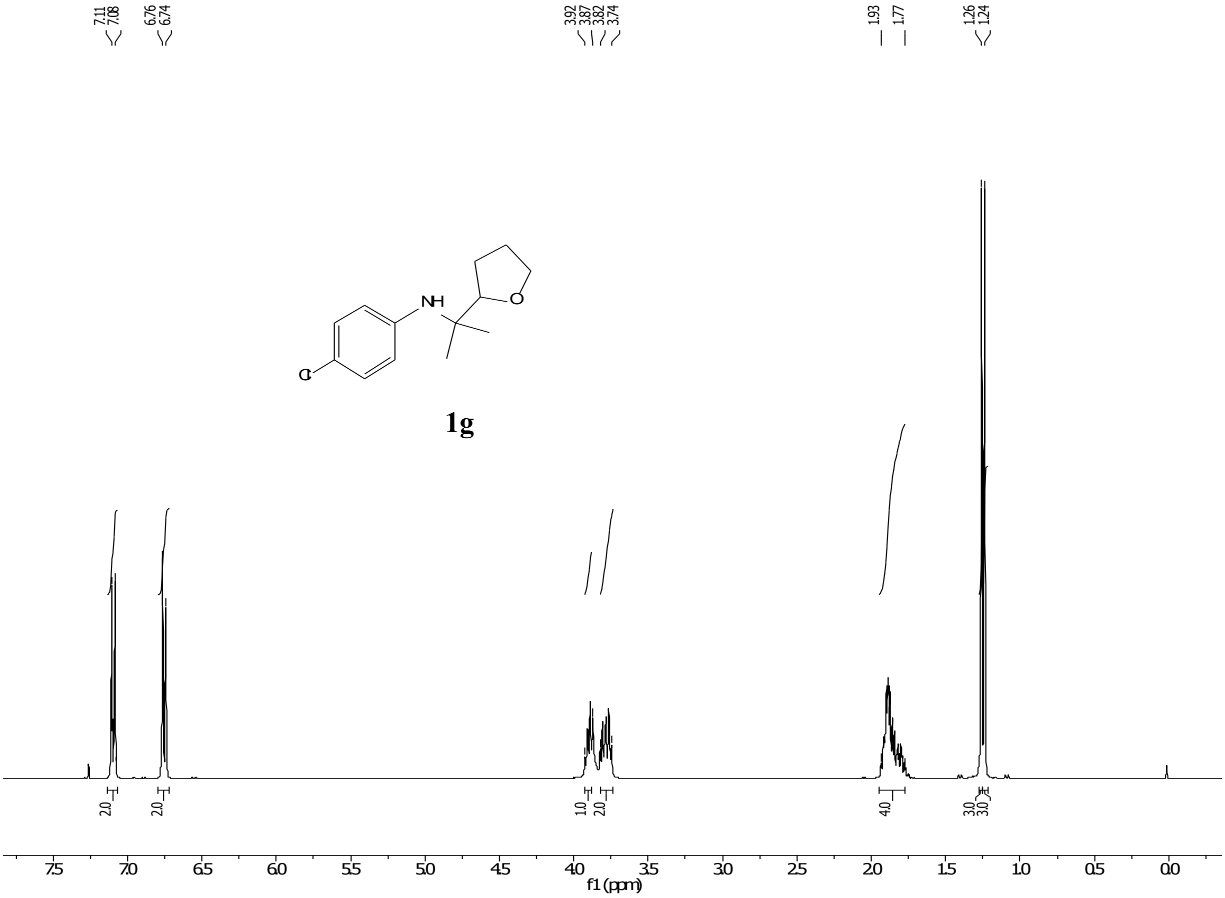
7.11
7.08
6.76
6.74
3.92
3.87
3.82
3.74
1.93
1.77
1.26
1.24
NH
O
Cl
2.0
2.0
1.0
2.0
4.0
3.0
3.0
7.5
7.0
6.5
6.0
5.5
5.0
4.5
4.0
3.5
3.0
2.5
2.0
1.5
1.0
0.5
0.0
f1 (ppm)

1g

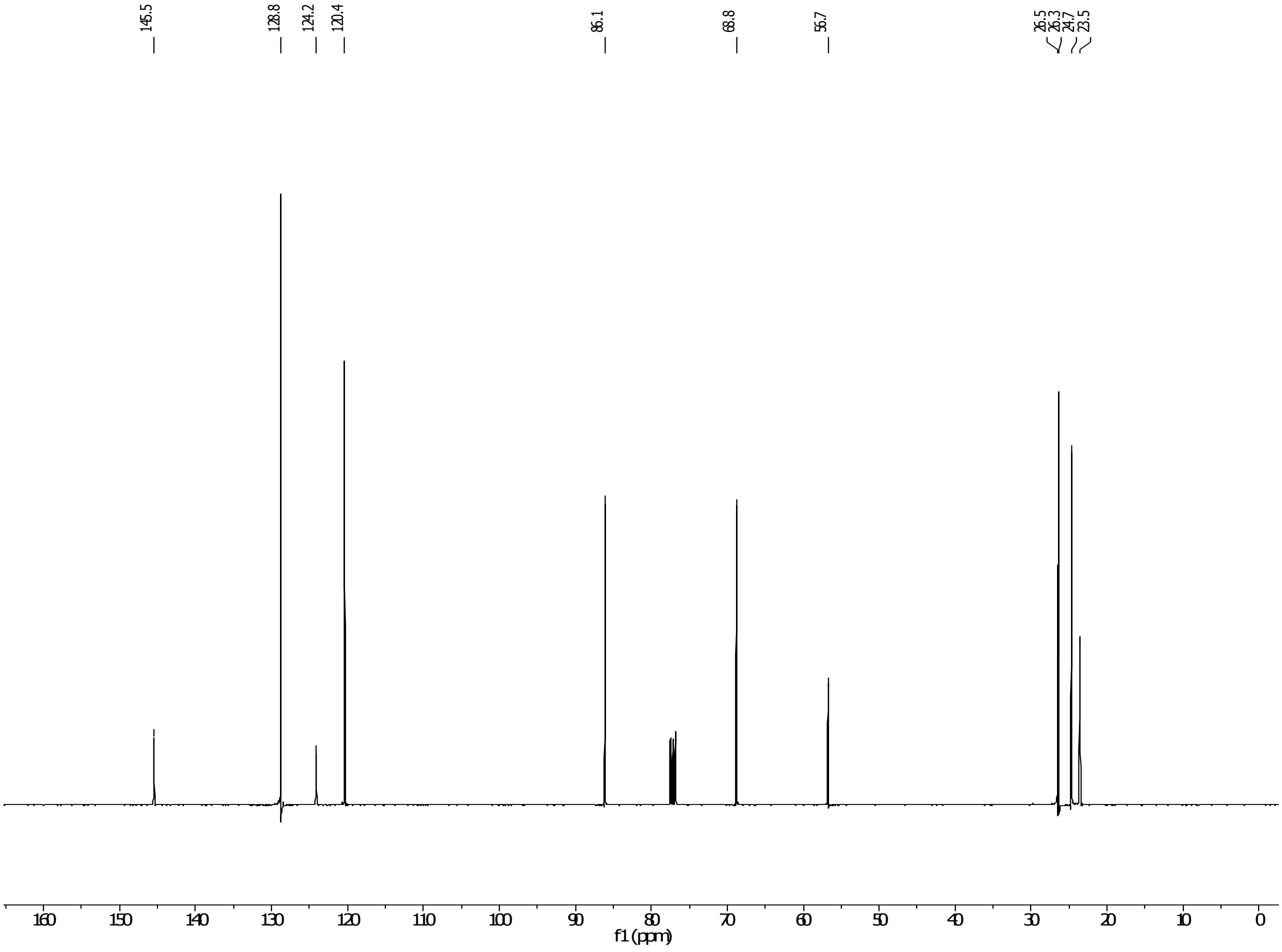
145.5
128.8
124.2
120.4
86.1
68.8
56.7
26.5
26.3
24.7
23.5
160
150
140
130
120
110
100
90
80
70
60
50
40
30
20
10
0
f1 (ppm)

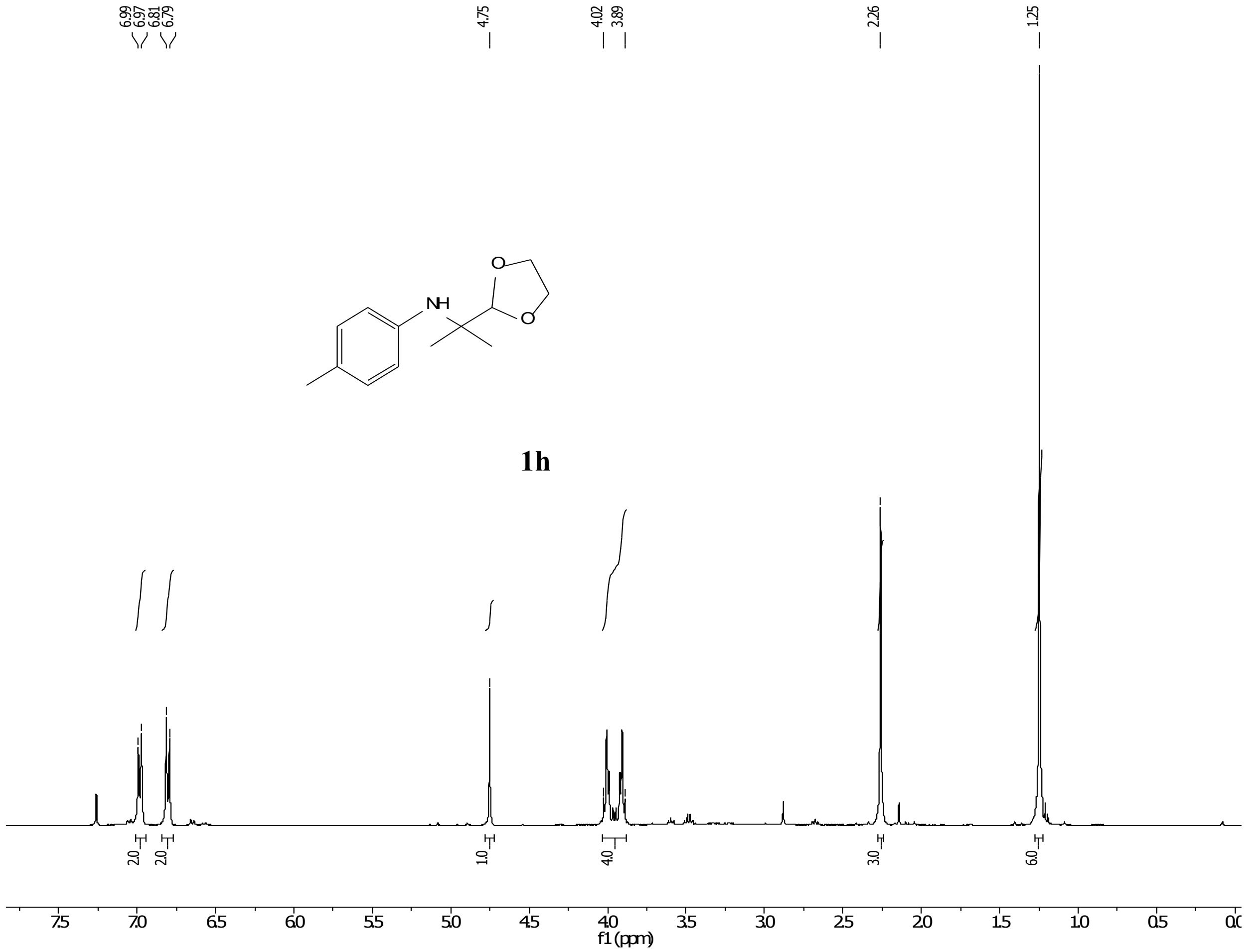
6.99
6.97
6.81
6.79
4.75
4.02
3.89
2.26
1.25
NH
O
O
1h
2.0
2.0
1.0
4.0
3.0
6.0
7.5 7.0 6.5 6.0 5.5 5.0 4.5 4.0 3.5 3.0 2.5 2.0 1.5 1.0 0.5 0.0
f1 (ppm)

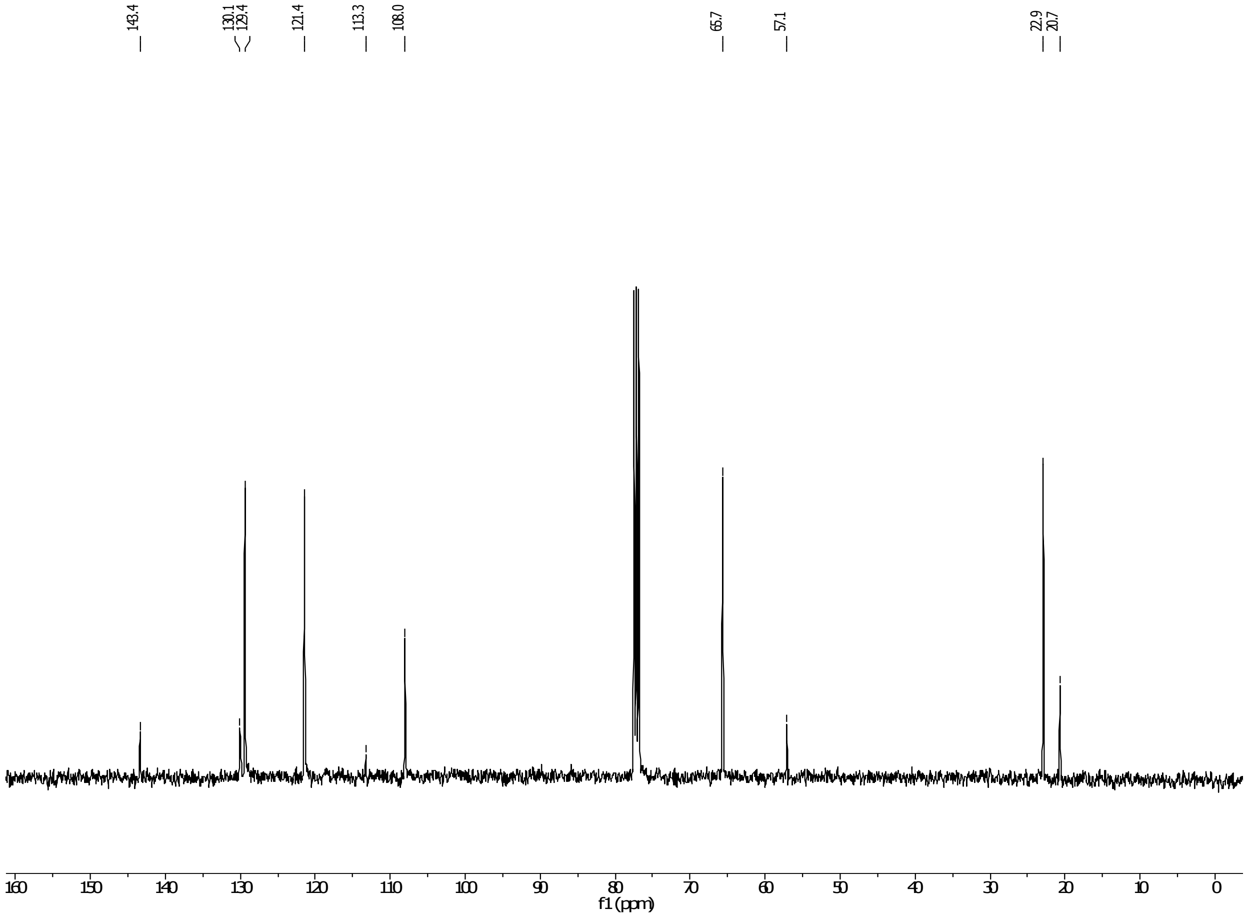
143.4
130.1
129.4
121.4
113.3
108.0
65.7
57.1
22.9
20.7
160 150 140 130 120 110 100 90 80 70 60 50 40 30 20 10 0
f1 (ppm)

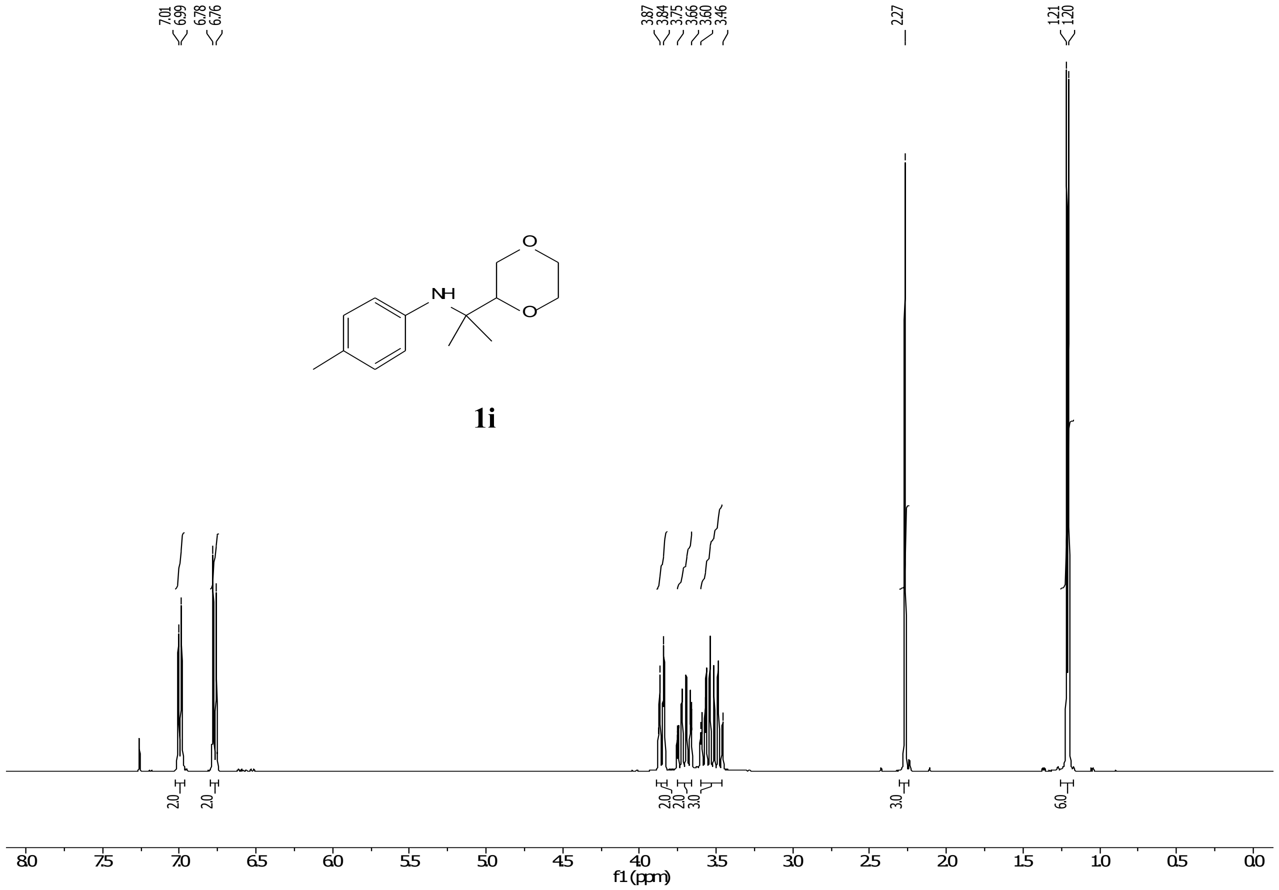
7.01
6.99
6.78
6.76
3.87
3.84
3.75
3.66
3.60
3.46
2.27
1.21
1.20
NH
O
O
1i
2.0
2.0
2.0
2.0
3.0
3.0
6.0
8.0 7.5 7.0 6.5 6.0 5.5 5.0 4.5 4.0 3.5 3.0 2.5 2.0 1.5 1.0 0.5 0.0
f1 (ppm)

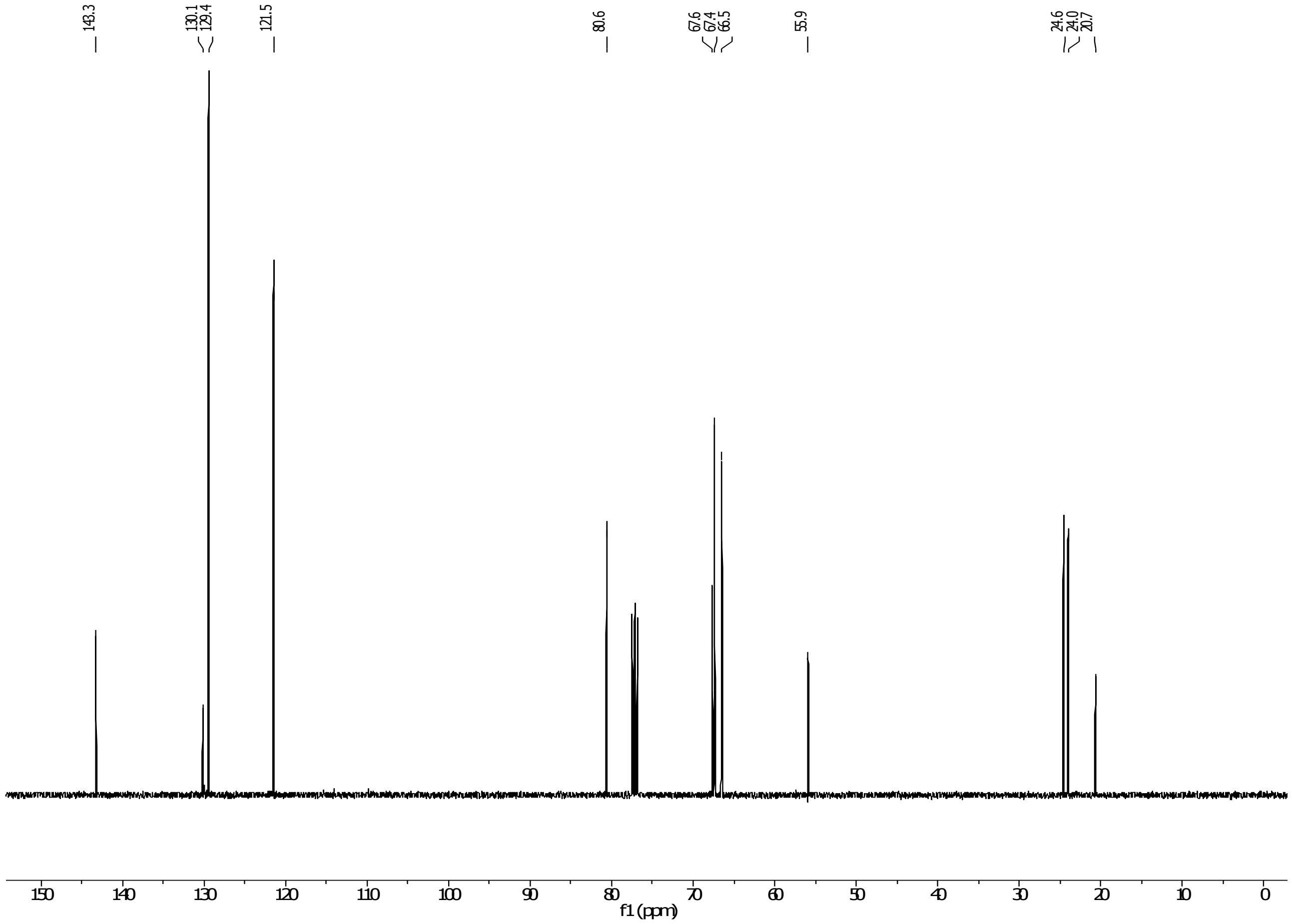
143.3
130.1
129.4
121.5
80.6
67.6
67.4
66.5
55.9
24.6
24.0
20.7
150 140 130 120 110 100 90 80 70 60 50 40 30 20 10 0
f1 (ppm)

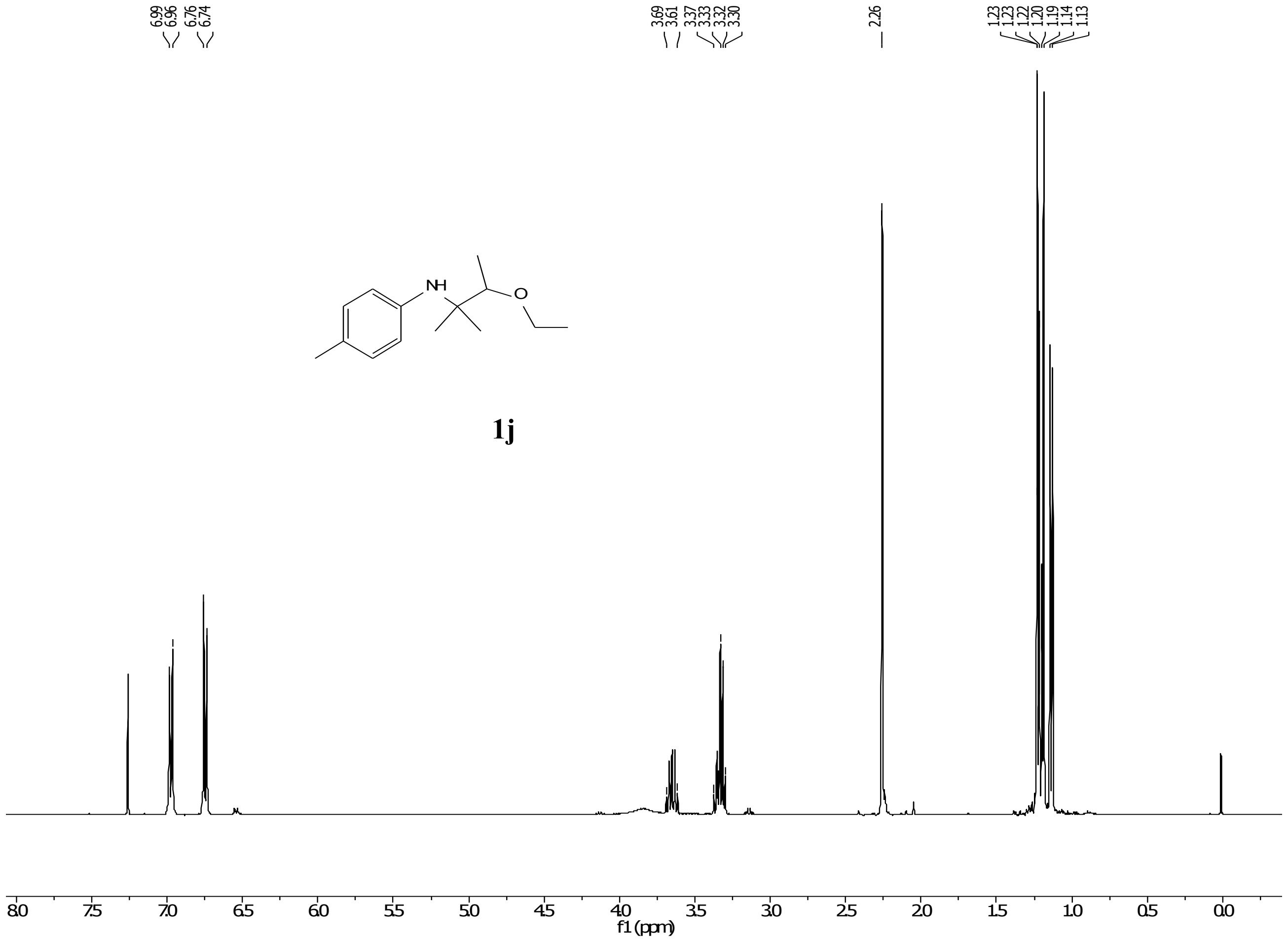
6.99
6.96
6.76
6.74
3.69
3.61
3.37
3.33
3.32
3.30
2.26
1.23
1.23
1.22
1.20
1.19
1.14
1.13
NH
O
1j
8.0 7.5 7.0 6.5 6.0 5.5 5.0 4.5 4.0 3.5 3.0 2.5 2.0 1.5 1.0 0.5 0.0
f1 (ppm)

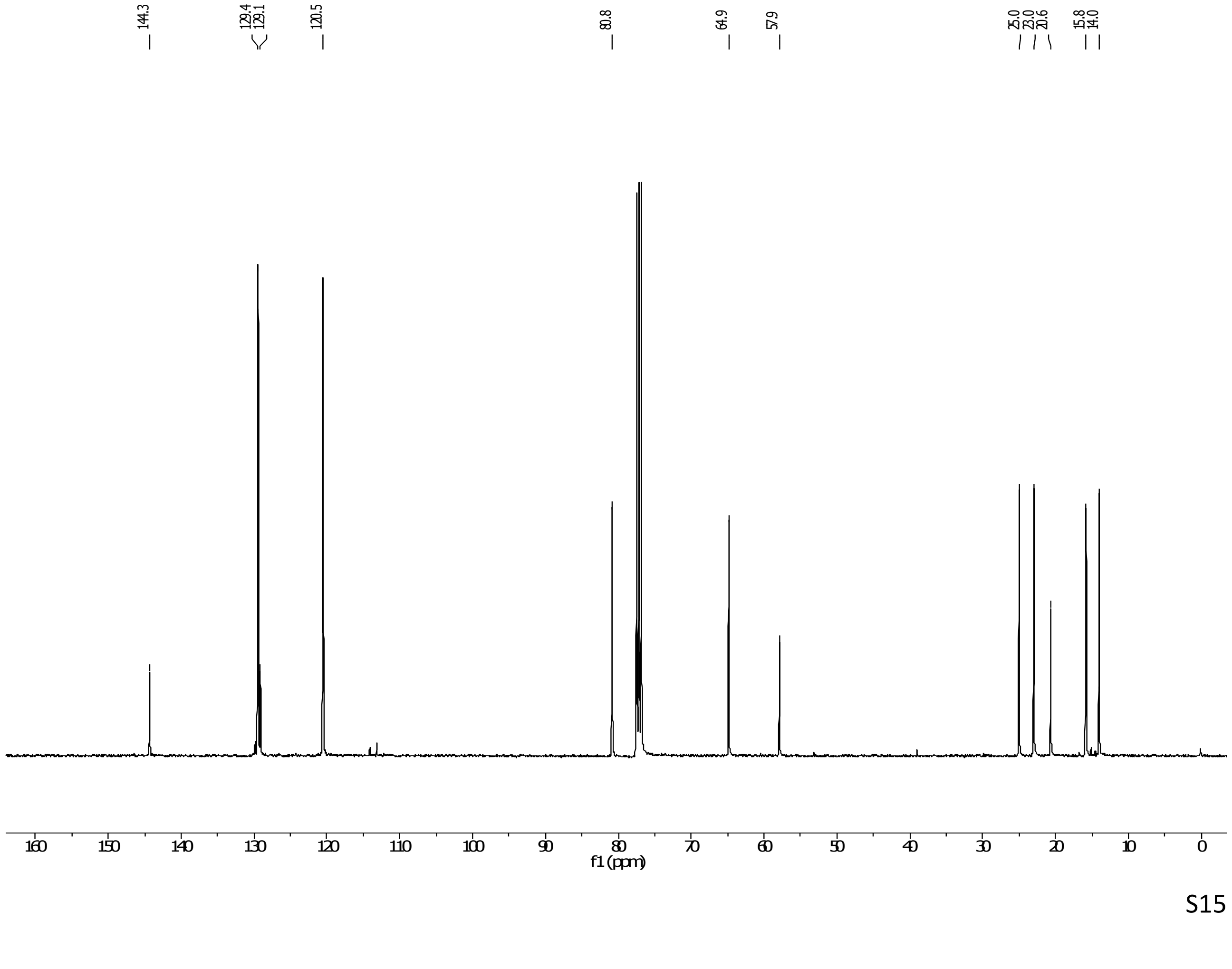
144.3
129.4
129.1
120.5
80.8
64.9
57.9
25.0
23.0
20.6
15.8
14.0
160 150 140 130 120 110 100 90 80 70 60 50 40 30 20 10 0
f1 (ppm)

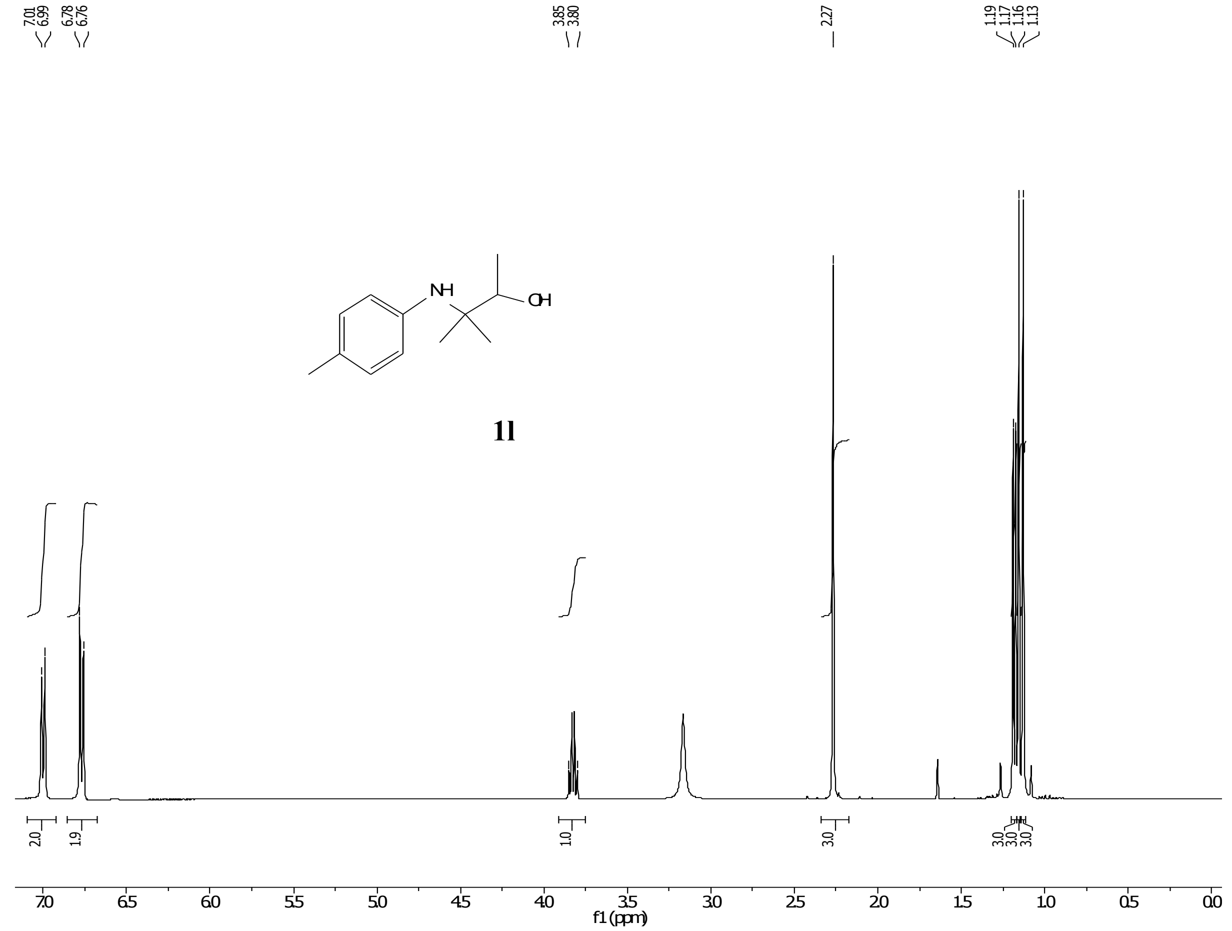
7.01
6.99
6.78
6.76
3.85
3.80
2.27
1.19
1.17
1.16
1.13
NH
OH
1l
2.0
1.9
1.0
3.0
3.0
3.0
3.0
f1 (ppm)

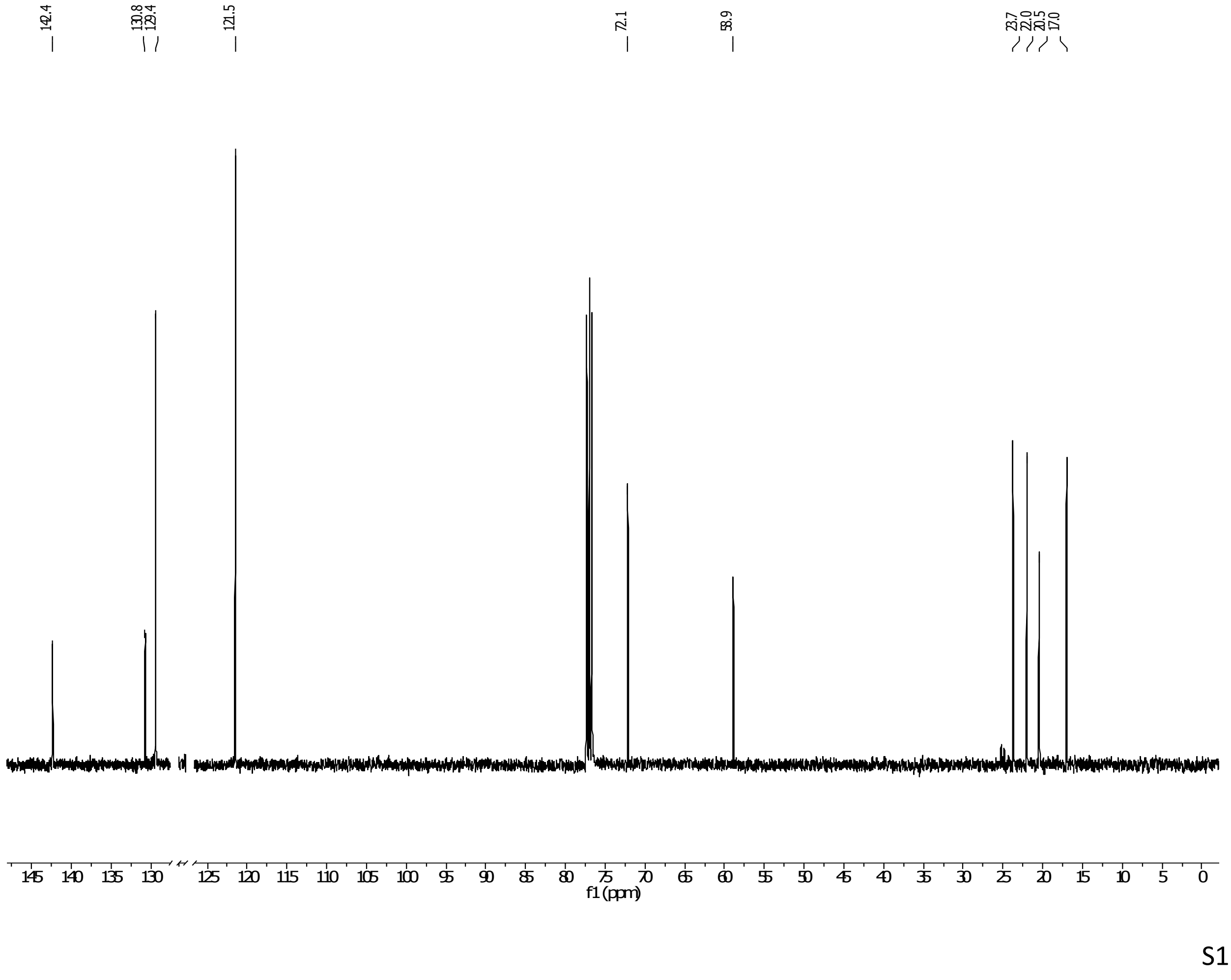
142.4
130.8
129.4
121.5
72.1
58.9
23.7
22.0
20.5
17.0
f1 (ppm)

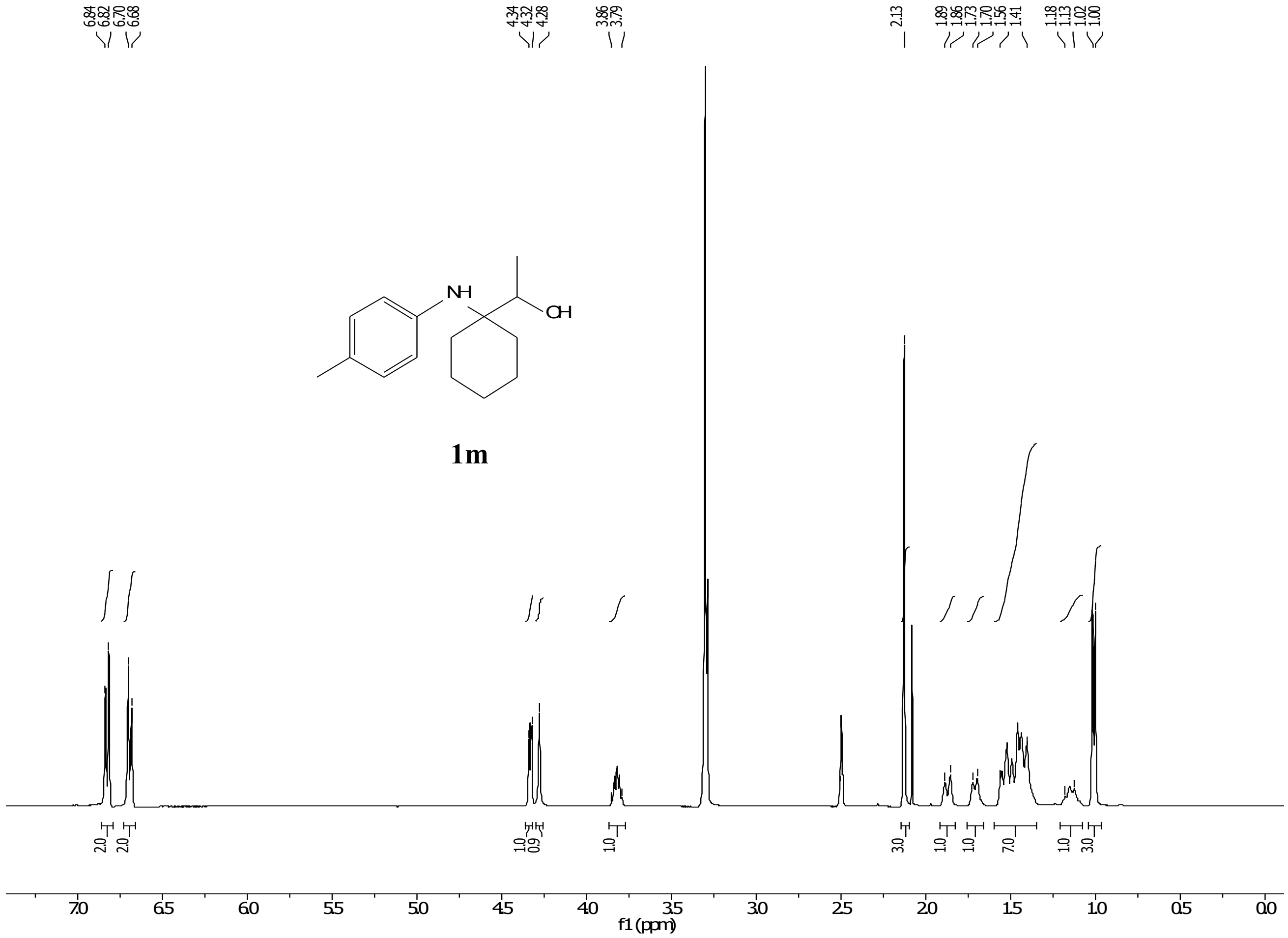
6.84
6.82
6.70
6.68
4.34
4.32
4.28
3.86
3.79
2.13
1.89
1.86
1.73
1.70
1.56
1.41
1.18
1.13
1.02
1.00
NH
OH
1m
2.0
2.0
1.0
0.9
1.0
3.0
1.0
1.0
7.0
1.0
3.0
f1 (ppm)

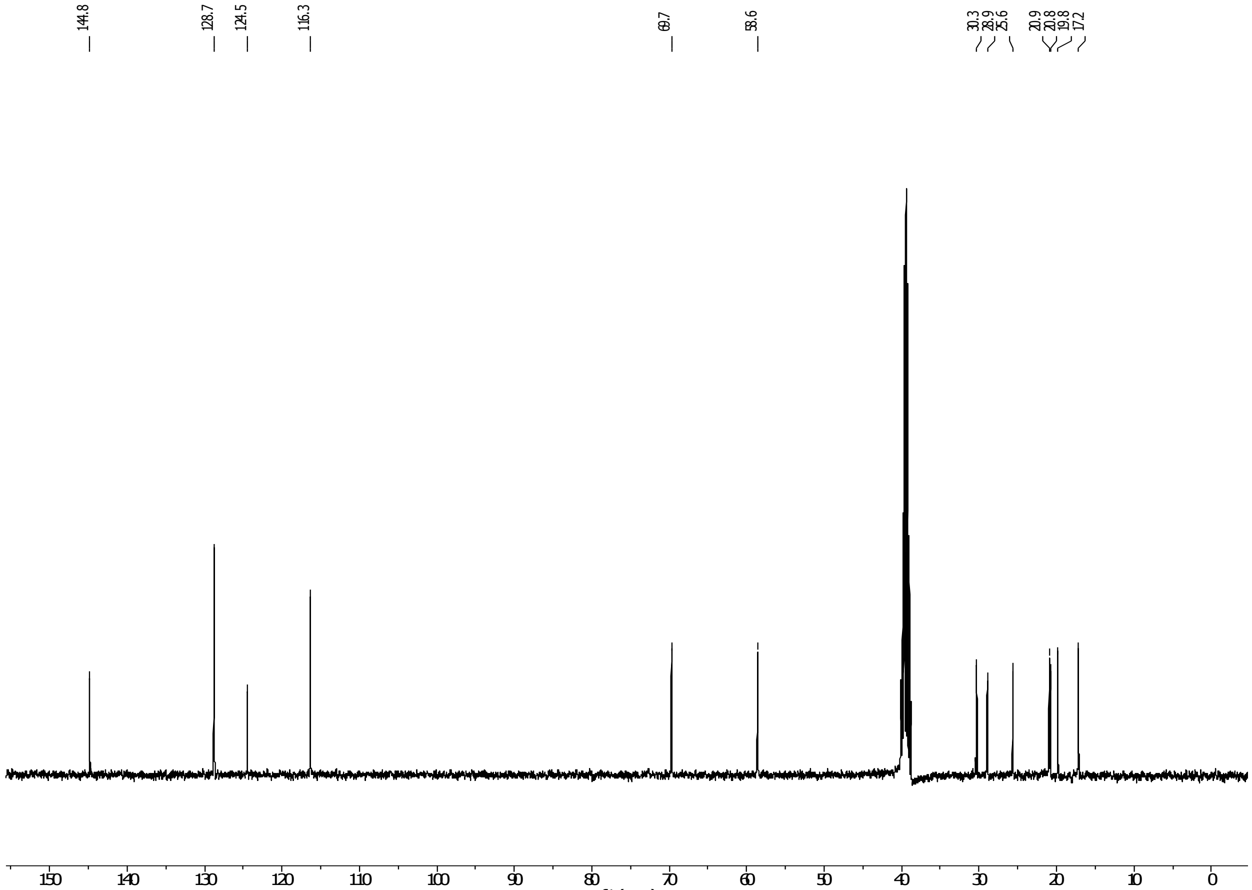
144.8
128.7
124.5
116.3
69.7
58.6
30.3
28.9
25.6
20.9
20.8
19.8
17.2
f1 (ppm)

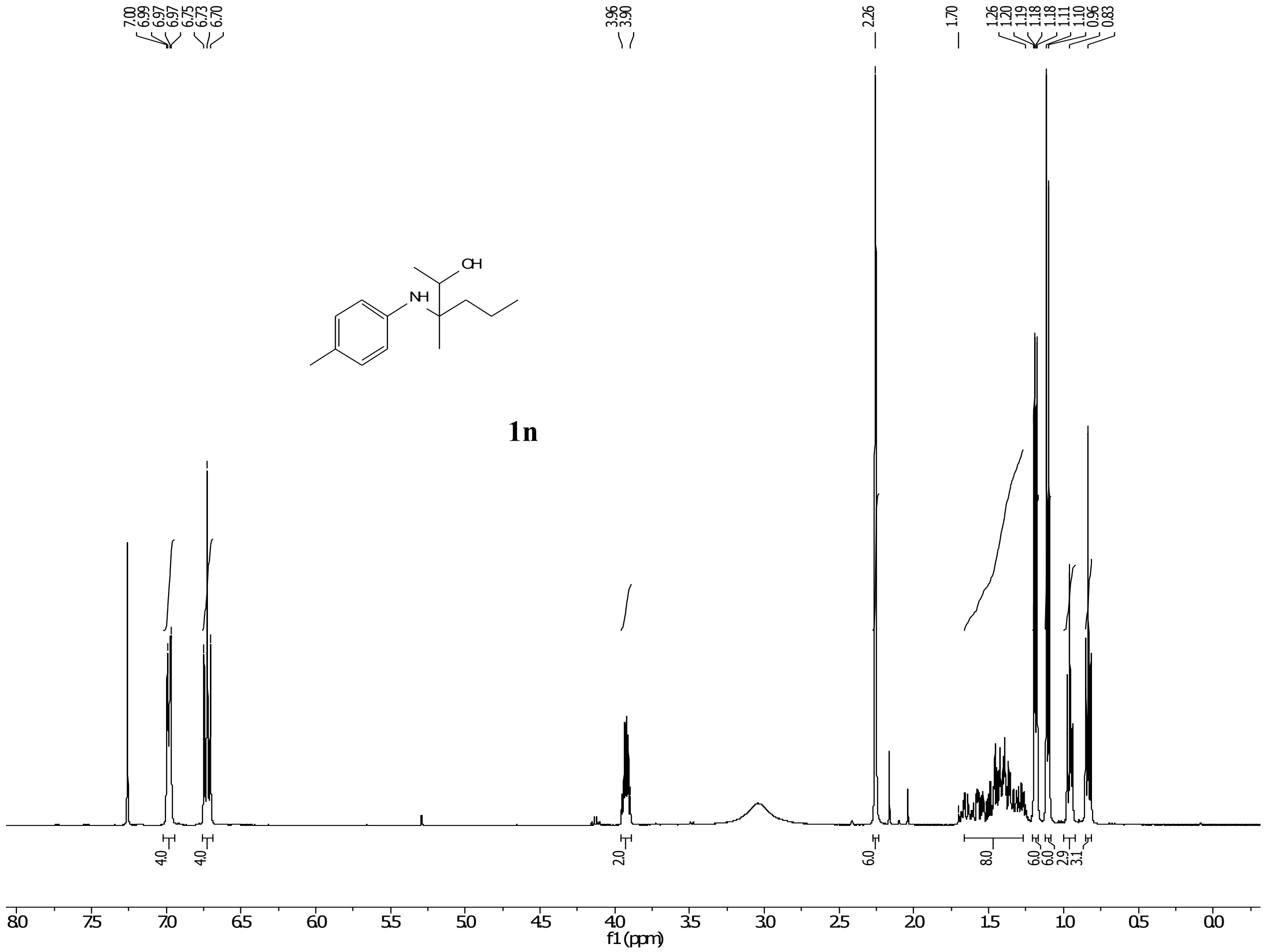

1n

7.00
6.99
6.97
6.97
6.75
6.73
6.70
3.96
3.90
2.26
1.70
1.26
1.20
1.19
1.18
1.18
1.11
1.10
0.96
0.83
4.0
4.0
2.0
6.0
8.0
6.0
6.0
2.9
3.1
8.0 7.5 7.0 6.5 6.0 5.5 5.0 4.5 4.0 3.5 3.0 2.5 2.0 1.5 1.0 0.5 0.0
f1 (ppm)

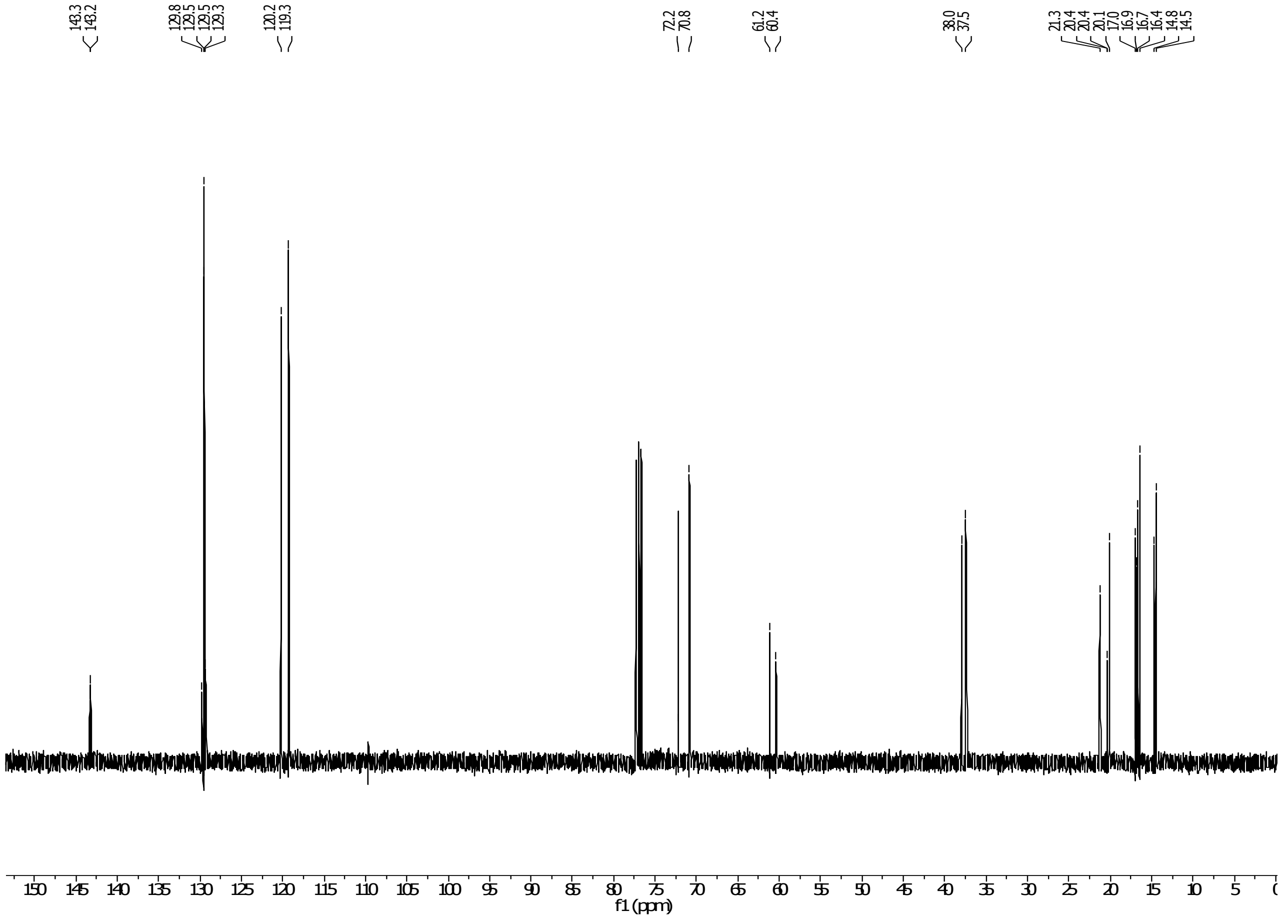

143.3
143.2
129.8
129.5
129.5
129.3
120.2
119.3
72.2
70.8
61.2
60.4
38.0
37.5
21.3
20.4
20.4
20.1
17.0
16.9
16.7
16.4
14.8
14.5
150 145 140 135 130 125 120 115 110 105 100 95 90 85 80 75 70 65 60 55 50 45 40 35 30 25 20 15 10 5
f1 (ppm)

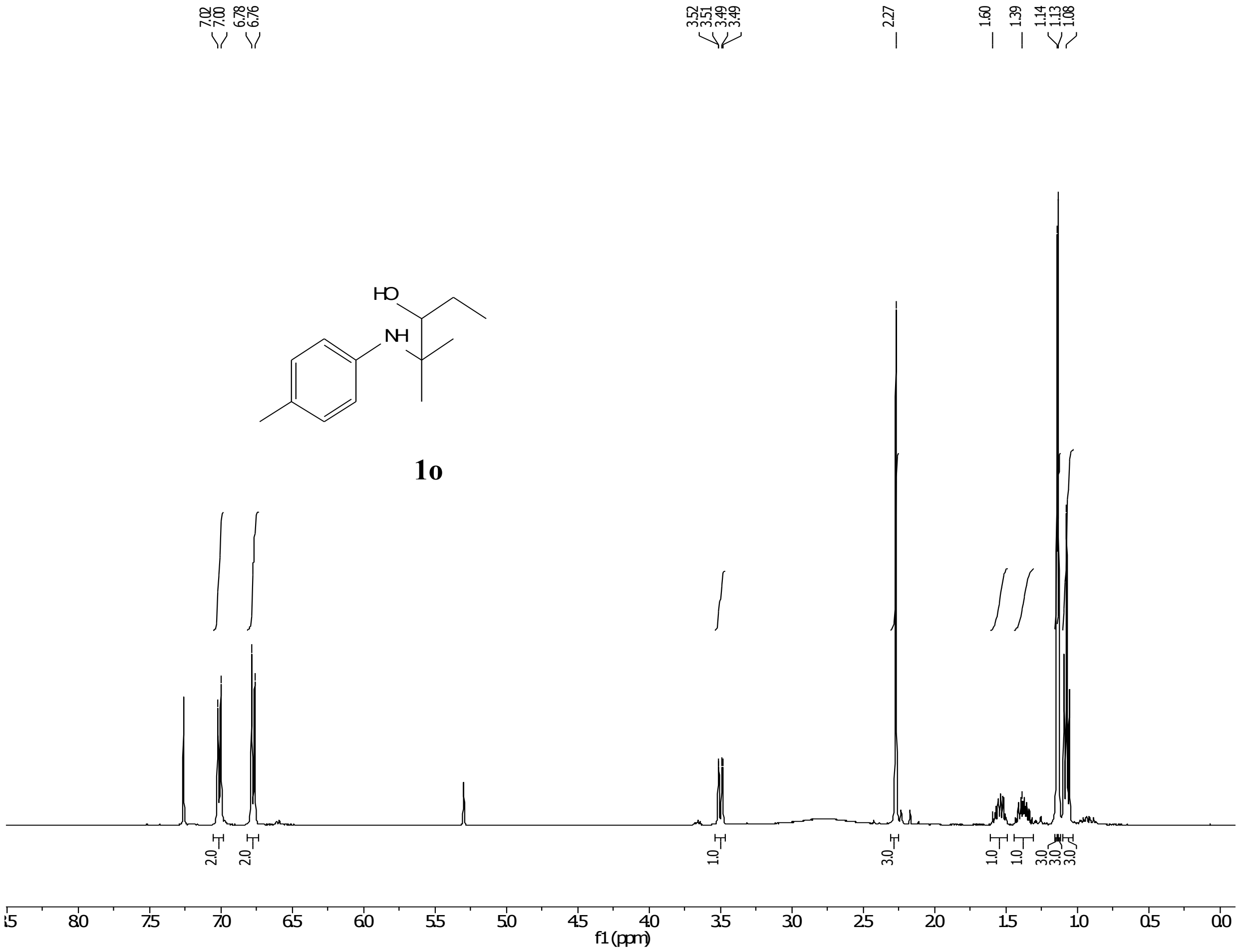
7.02
7.00
6.78
6.76
3.52
3.51
3.49
3.49
2.27
1.60
1.39
1.14
1.13
1.08
HO
NH
1o
2.0
2.0
1.0
3.0
1.0
1.0
3.0
3.0
3.0
f1 (ppm)

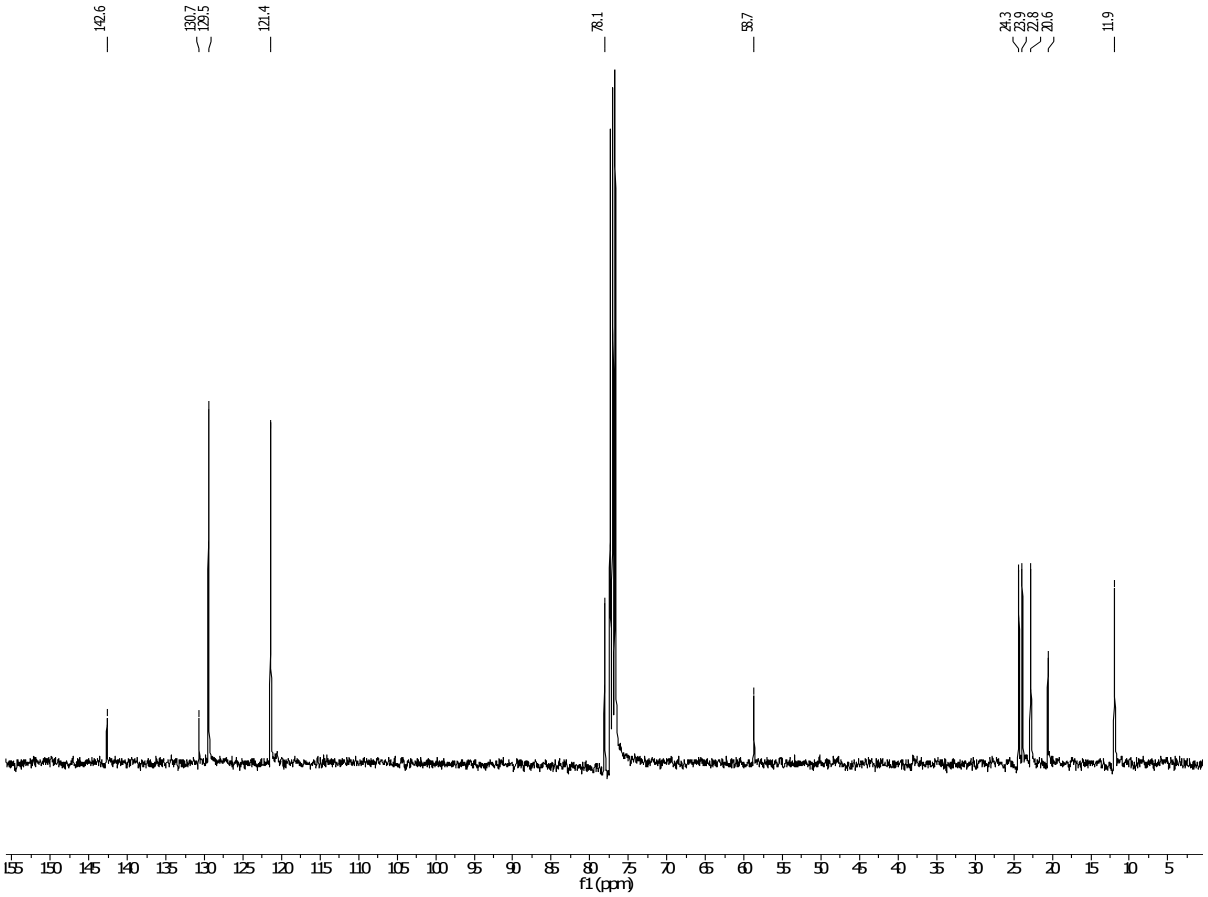
142.6
130.7
129.5
121.4
78.1
58.7
24.3
23.9
22.8
20.6
11.9
f1 (ppm)

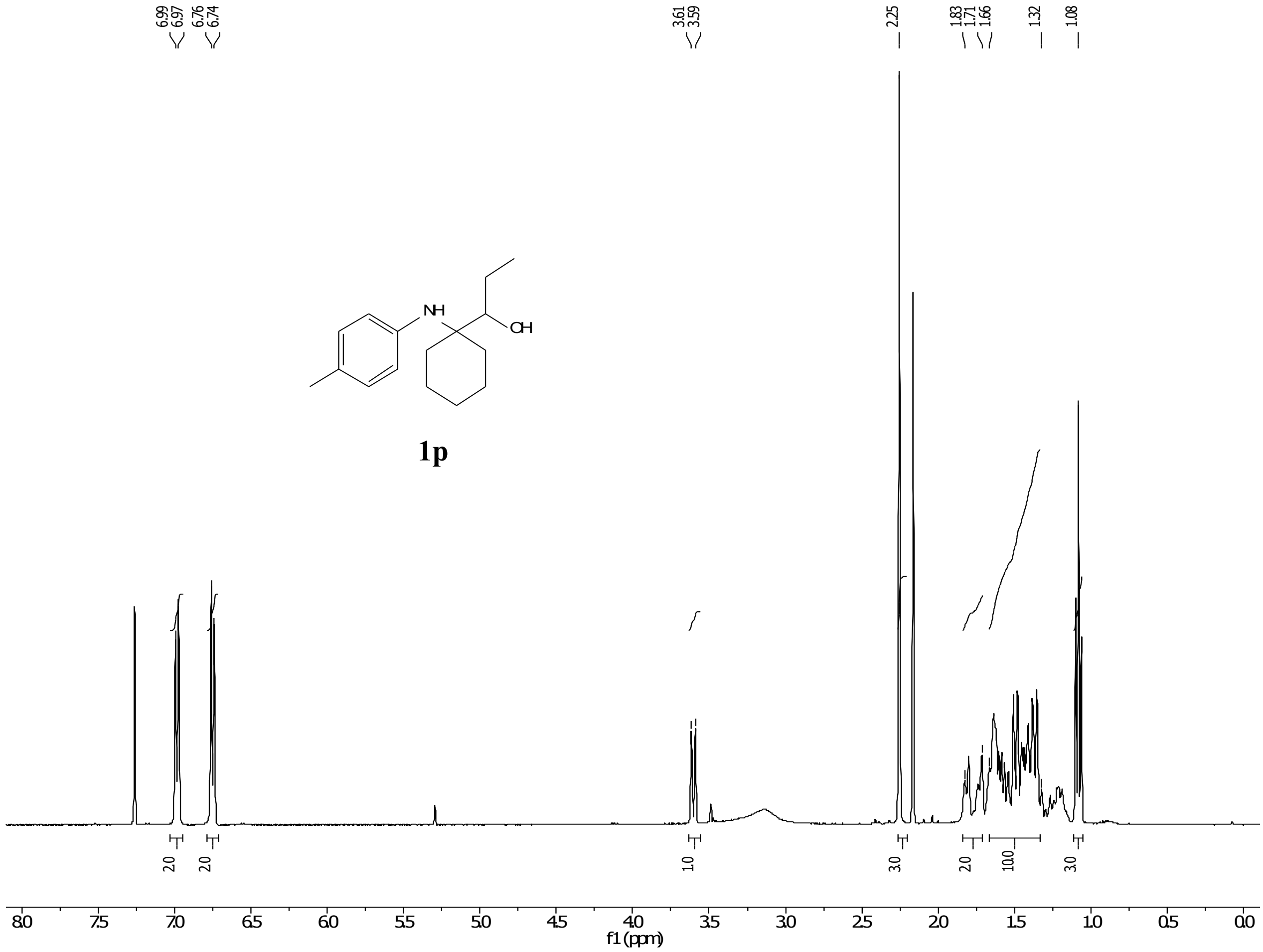
6.99
6.97
6.76
6.74
3.61
3.59
2.25
1.83
1.71
1.66
1.32
1.08
NH
OH
1p
2.0
2.0
1.0
3.0
2.0
10.0
3.0
8.0 7.5 7.0 6.5 6.0 5.5 5.0 4.5 4.0 3.5 3.0 2.5 2.0 1.5 1.0 0.5 0.0
f1 (ppm)

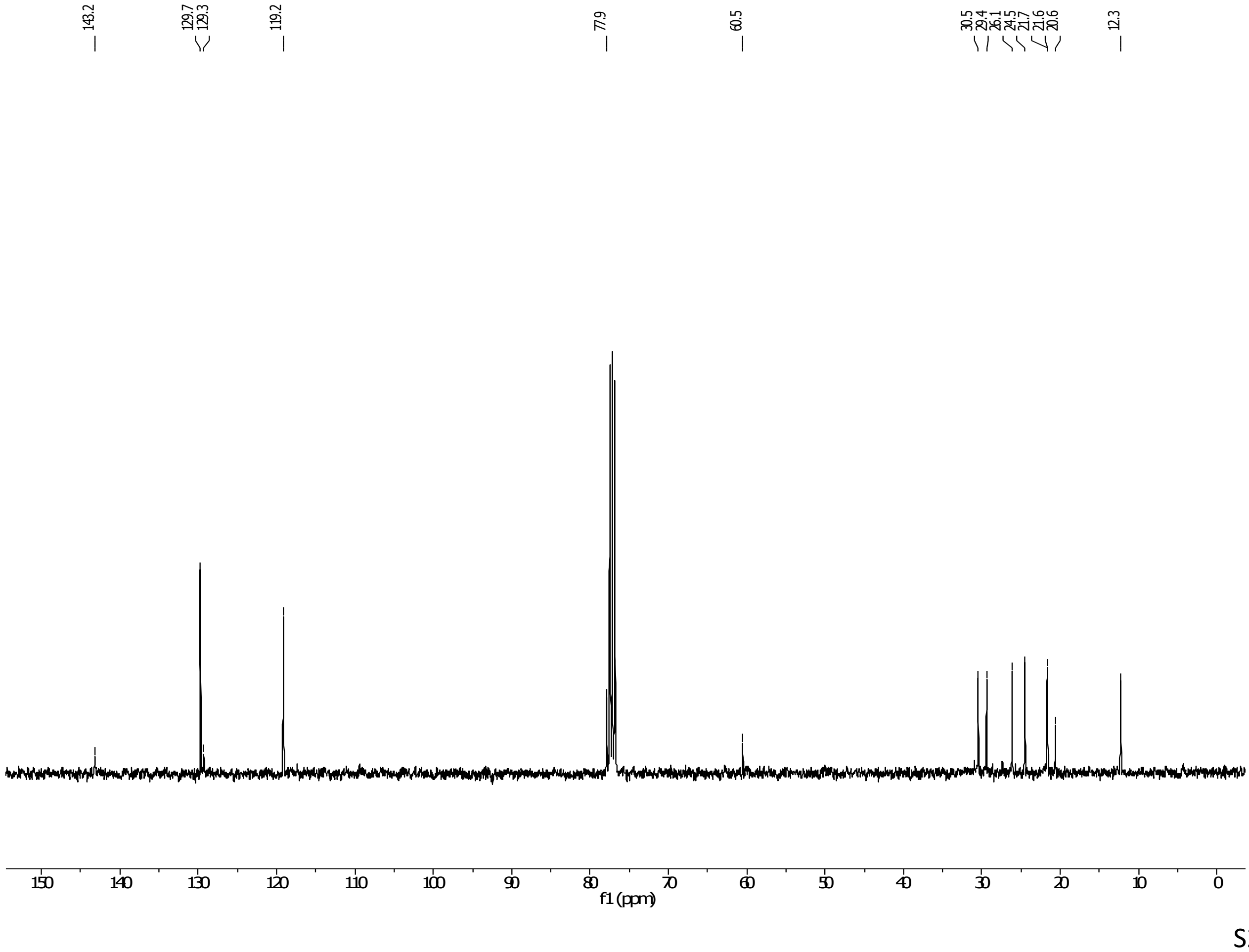
143.2
129.7
129.3
119.2
77.9
60.5
30.5
29.4
26.1
24.5
21.7
21.6
20.6
12.3
150 140 130 120 110 100 90 80 70 60 50 40 30 20 10 0
f1 (ppm)
